# CO_2_ Conversion by a Metal-Coordinated Single Amino Acid Carbonic Anhydrase Enzyme Mimic

**DOI:** 10.1021/acsami.5c26143

**Published:** 2026-03-26

**Authors:** Adithya Ramesh, Xiaoyu Wang, Subrat Vishwakarma, Oren Ben-Zvi, Linda J. W. Shimon, Sigal Rencus-Lazar, Pandeeswar Makam, Hao Dong, Ehud Gazit, Om Shanker Tiwari, Brian A. Rosen

**Affiliations:** † Department of Chemistry, 28673 National Institute of Technology Warangal, Hanamkonda 506004, Telangana India; ‡ State Key Laboratory of Analytical Chemistry for Life Science, Kuang Yaming Honors School, Chemistry and Biomedicine Innovation Center (ChemBIC), Institute for Brain Sciences, 12581 Nanjing University, Nanjing 210023, China; § Department of Chemistry, 79203 Indian Institute of Technology (BHU), Varanasi, UP 221005, India; ∥ School of Plant Sciences and Food Security, The George S. Wise Faculty of Life Sciences, 26745 Tel Aviv University, Tel Aviv, Israel; ⊥ Department of Chemical Research Support, Weizmann Institute of Science, Rehovot 7610001, Israel; # The Shmunis School of Biomedicine and Cancer Research, The George S. Wise Faculty of Life Sciences, Tel Aviv University, Tel Aviv 6997801, Israel; ∇ Department of Materials Science and Engineering, Tel Aviv University, Tel Aviv 6997801, Israel

**Keywords:** CO_2_ sequestration, carbonic anhydrase, bionanozyme, catalyst, supramolecular amyloid, environmental sustainability, *ab initio* calculations, reaction mechanism

## Abstract

The rising atmospheric concentration of carbon dioxide (CO_2_) is assumed to be a key factor in global climate change, requiring robust and sustainable carbon conversion technologies. While carbonic anhydrase (CA) is a highly efficient enzyme for CO_2_ sequestration, its industrial application is limited by stability, cost, and scalability challenges. To address these limitations, we developed a CA-mimetic metal-amino acid (Phe-Zn­(II)) bionanozyme featuring amyloid-like supramolecular cross-β-sheet architecture that provides high structural stability and recyclability. Gas chromatography (GC) analysis of a continuous flow bubble reactor charged with Phe-Zn­(II) bionanozyme exhibits a CO_2_ conversion efficiency of approximately 18% in an aqueous medium (pH 7.0, 25 °C, ambient pressure), while maintaining remarkable structural integrity as confirmed by postcatalysis PXRD analysis. The amyloid-like supramolecular cross-β-sheet architecture, stabilized by π-π stacking and intermolecular hydrogen bonding, generates a confined catalytic microenvironment that enhances Zn­(II) Lewis’s acidity and promotes efficient CO_2_ hydration, which is crucial compared to previous reports. Next, density functional theory (DFT) calculations reveal a three-step catalytic pathway involving hydroxide ion generation, nucleophilic attack, and carbonic acid formation, with a rate-determining barrier of 12.3 kcal/mol, making the reaction feasible at room temperature. We also investigated the impact of different amino acids coordinated with Zn, finding that Phe-Zn­(II) shows higher catalytic activity. This is due to the stronger electron-withdrawing effect of the phenyl group, which enhances the Lewis acidity of Zn^2+^, activates the Zn^2+^–OH_2_ bond, and lowers the rate-determining barrier. Taken together, the combination of experimental catalysis, structural robustness, and mechanistic validation highlights Phe-Zn­(II) as a promising, cost-effective, and minimalistic catalyst yet efficient carbonic anhydrase mimic for CO_2_ conversion, paving the way for scalable and sustainable carbon sequestration strategies critical for mitigating climate change.

## Graphical abstract

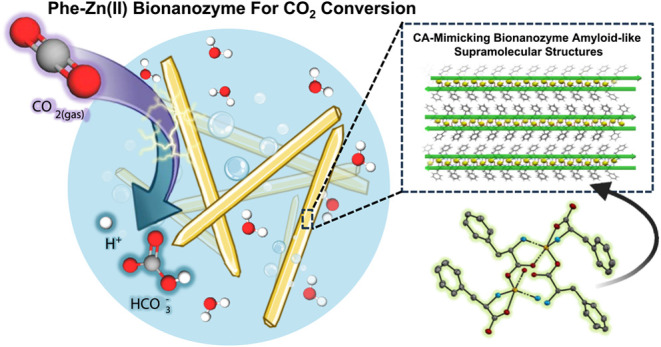

## 1 Introduction

The unregulated release of carbon dioxide (CO_2_) into the atmosphere, primarily driven by industrial activities such as power generation, extensive automobile usage, and the combustion of fossil fuels, has escalated significantly in recent decades.^1–3^ This trend has positioned CO_2_ emissions as a predominant contributor to the greenhouse effect, thereby exacerbating global warming and catalyzing long-term climate change.^4^ Given the profound environmental and socio-economic ramifications of this phenomenon, it is imperative to implement stringent measures aimed at curbing excessive CO_2_ emissions. Thus, extensive research and development efforts have been undertaken globally to address the impacts of increasing CO_2_ levels through carbon conversion and storage strategies. In this direction, the development and deployment of advanced technologies for CO_2_ conversion, utilization, and sequestration (CCUS) represent a promising avenue for mitigating atmospheric CO_2_ concentrations.^5^ These strategies encompass physical, chemical, and biological approaches, representing the most prevalent methods for CO_2_ conversion.^6–9^ Physical techniques rely on the adsorption of CO_2_ onto porous materials or its absorption in the liquid phase, often necessitating high-purity CO_2_. Despite their simplicity, these methods are characterized by relatively modest removal efficiencies. Chemical methods, by contrast, leverage reactions between absorbents and CO_2_, transforming the gas into valuable chemical products.^10–12^ These methods offer the advantage of higher removal efficiency, with high-purity CO_2_ recoverable through desorption processes. However, the absorbents, which often contain strong alkaline compounds present challenges, including potential equipment corrosion and the risk of secondary environmental pollution if not properly managed.^13^

Chemical absorption using amine-based absorbents stands as the most widely adopted and recognized method for postcombustion CO_2_ conversion. Renowned for its high efficiency, cost-effectiveness, and technological maturity, this approach remains a cornerstone in CO_2_ mitigation strategies.^14,15^ Despite their widespread use, amine-based absorbents exhibit significant limitations. These include high energy and electricity demands, CO_2_ emissions during their production, toxicity, and the generation of degradation byproducts. Moreover, their volatility and corrosive nature pose operational challenges. Regeneration of the solvent and recovery of the converted CO_2_ further necessitate substantial energy input, adding to the overall process complexity and cost.^16,17^

A promising solution to these challenges lies in the use of the enzyme carbonic anhydrase (CA), which, with a Zn^2+^ ion at its active site, catalyzes the reversible hydration of CO_2_ to bicarbonate in aqueous media at pH 7 with remarkable efficiency. CA is a zinc-containing metalloenzyme catalyst found across diverse organisms, including mammals, plants, algae, archaea, vertebrates, and bacteria.^18–21^ It plays a critical role in regulating essential biological processes, making it an attractive candidate for CO_2_ conversion technologies.^22–25^ CA supports metabolic pathways involving dissolved CO_2_ (or its hydrated form as bicarbonate) and helps maintain cellular pH balance through bicarbonate buffering.^26,27^ However, the CA application faces challenges, including limited stability under high temperatures (as in flue gas), alkaline pH, and high salt concentrations during absorption. High production costs, difficulties in recovery and reuse, and susceptibility to inhibition by impurities such as heavy metals, SO_*x*_, and NO_*x*_ further constrain its utility in industrial settings.^28,29^

To address the limitations associated with carbonic anhydrase (CA), the development of robust CA-mimetic biomaterials has emerged as a key focus of ongoing research. These efforts aim to replicate the catalytic efficiency of natural CA while enhancing stability and resilience under industrial conditions, such as high temperatures, alkaline pH, and high salt concentrations.^30^ Researchers are exploring innovative strategies to design and fabricate artificial CA mimics that can withstand harsh environments, reduce production costs, and improve recovery and reusability.^31^ Additionally, these biomimetic systems are being tailored to resist inhibition by flue gas impurities such as heavy metals, SO_*x*_, and NO_*x*_, paving the way for more efficient and sustainable CO_2_ conversion technologies. The CA-mimicking structure showcased exceptional biocatalytic performance in both ester hydrolysis and CO_2_ hydration in an aqueous medium. Its remarkable stability further highlights its potential as a biobased, high-performance sorbent for carbon conversion applications.^32–36^ Various strategies are being pursued to mimic the catalytic activity of CAs, including zeolites,^37,38^ metal–organic frameworks,^39–41^ metal complexes,^42^ and polymers.^43^ However, these artificial systems often diverge significantly from the structure and functionality of native enzymes.

Among artificial CA mimetics, biomolecular assemblies offer a promising alternative, providing a supramolecular framework for constructing artificial enzymes.^44^ These assemblies rely on noncovalent interactions among their building blocks mediating self-assembly processes, thus closely mimicking the architecture and dynamics of natural proteins. David S. Eisenberg and co-workers have demonstrated that amyloid self-assembling protein fibers hold significant potential for selective CO_2_ conversion.^45^ These protein-based structures naturally form through the self-assembly of peptide chains into highly ordered fibrous aggregates, exhibiting remarkable stability and surface functionality. Their unique structural and chemical properties enable efficient CO_2_ conversion, making them an attractive platform for developing sustainable and biocompatible solutions for carbon management.^46–48^ However, the synthesis of these materials often requires precise control over peptide sequence, concentration, and environmental conditions to ensure efficient self-assembly into functional fibers. Additionally, the long-term stability of amyloid-based materials can be compromised by factors such as high temperature, pH variations, or the presence of certain contaminants, which can lead to degradation or loss of activity. Next, Zn^2+^-coordinated peptide amyloid assemblies have been utilized for CO_2_ conversion, offering enhanced catalytic activity and stability due to zinc ion complexation. These assemblies facilitate ester bond hydrolysis, a typical reaction of CAs, highlighting their potential as efficient, stable biomimetic catalysts for CO_2_ conversion and related applications.^49–51^ Interestingly, the Zn^2+^ ion serves as a crucial cofactor, while the amyloid cross-β-sheet folding provides an optimal structural scaffold for designing artificial CA mimetics. Furthermore, aromatic dipeptides and metabolites show promise as minimalistic mimics of amyloid assemblies, offering a simplified yet effective approach to replicating the catalytic functions of natural CAs.^52^ Due to their strong tendency toward the formation of strong intermolecular interactions, amino acid–based complexes with metal ions demonstrate exceptional properties such as a remarkable framework of crystal structure with good mechanical strength. The main drawbacks associated with dipeptides are the difficult synthesis procedure and the challenge of controlling the sizes of assemblies.^53^ Our lab subsequently developed a minimalistic, amyloid-inspired assembly referred to as a ‘bionanozyme’. A bionanozyme refers to a bioinspired nanoscale supramolecular assembly that exhibits enzyme-mimetic catalytic activity. This innovative structure is composed of the single amino acid phenylalanine (Phe) in combination with Zn­(II) ions, resulting in assemblies characterized by a highly ordered and perfect crystalline architecture of Phe-Zn­(II) assemblies, which function as a highly efficient CA-mimic.^29^ Phenylalanine (Phe) was selected as the ligand scaffold due to its intrinsic propensity to self-assemble into amyloid-like cross-β-sheet architectures, enabling the formation of an ordered supramolecular framework rather than discrete coordination complexes. In contrast to simple Zn-amino acid systems that generate isolated molecular species, the Phe-Zn­(II) assembly forms a hierarchical crystalline structure stabilized by π-π stacking interactions and hydrogen bonding. This supramolecular organization creates a confined microenvironment capable of modulating local hydrophobicity and stabilizing catalytic intermediates. Furthermore, the aromatic phenyl side chain enhances the electron-withdrawing environment around the Zn­(II) center, increasing its Lewis acidity and facilitating Zn–OH_2_ bond activation. This combination of structural hierarchy and electronic modulation distinguishes Phe-Zn­(II) from conventional Zn-ligand complexes and underpins its catalytic performance. This innovative design demonstrates exceptional CO_2_ to bicarbonate conversion efficiency, coupled with remarkable robustness, making it a promising candidate for CO_2_ conversion applications.

In this study, we developed an advanced catalytic methodology to enhance the efficiency and environmental sustainability of CO_2_ conversion in aqueous media. The research focused on the use of Phe-Zn­(II) with another aromatic amino acid containing Trp-Zn­(II) (tryptophan-zinc) crystal, with the help of both synthesized compounds, we have investigated the role of aromaticity in influencing the CO_2_ conversion process. Colorless rod-like crystals of Phe-Zn­(II) were grown and subjected to comprehensive characterization. Detailed structural analysis was performed using single-crystal X-ray diffraction (SC-XRD) and powder X-ray diffraction (PXRD), providing insight into the crystalline framework and structural integrity. Optical microscopy facilitated the examination of morphological features, while high-resolution scanning electron microscopy (HR-SEM) and transmission electron microscopy (TEM) enabled a detailed investigation of surface topography and nanostructural attributes. Subsequently, energy-dispersive X-ray spectroscopy (EDX) was employed to confirm the elemental composition and the incorporation of zinc within the supramolecular architectures. Additional analyses included mass spectrometry for molecular-level insights and X-ray photoelectron spectroscopy (XPS) to elucidate surface chemical states and binding environments. We report the rapid formation of Phe-Zn­(II) single crystals in the form of supramolecular amyloid-like cross-β-sheet assemblies. Using a combination of X-ray diffraction and spectroscopic techniques, we characterized the molecular packing interactions between Phe and Zn­(II) ions. The combined results not only validated the presence of Zn within the Phe-Zn­(II) structures but also provided a robust framework for understanding the interaction dynamics of these materials with CO_2_, thereby offering valuable insights into their catalytic performance in eco-friendly CO_2_ conversion processes (Figure 1). To gain a deeper understanding of the catalytic mechanism, we employed *ab initio* calculations based on the crystal structure of Phe-Zn­(II) to investigate the CO_2_ hydration process. The nucleophilic attack of hydroxide ion (OH^–^) on the electrophilic carbon atom of CO_2_ was identified as the rate-determining step (RDS). By comparing the coordination effects of different amino acids (Phe and Trp) with Zn­(II), our results indicate that the stronger electron-withdrawing effect of the phenyl group endows Phe-Zn­(II) with higher catalytic activity.

**Figure 1:**
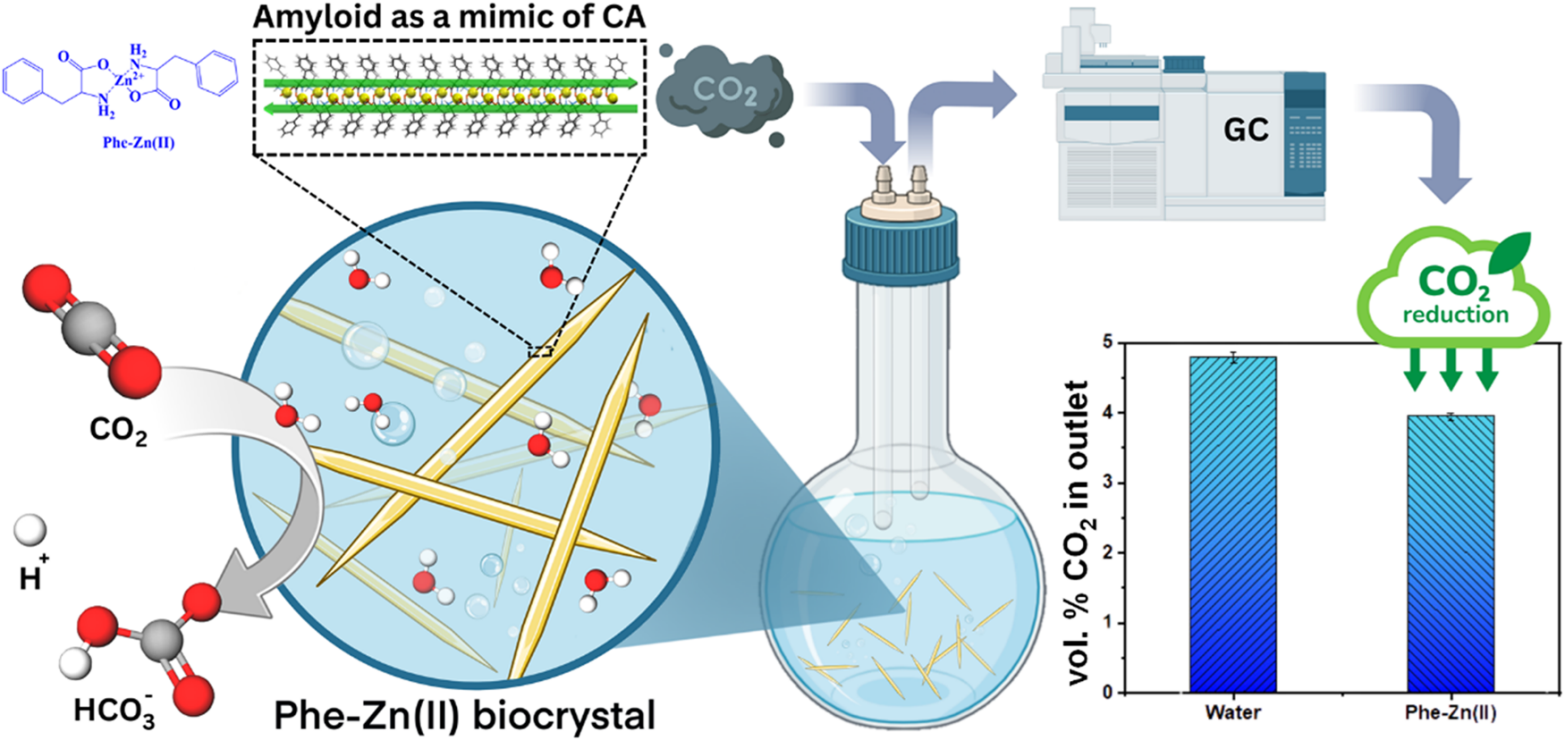
Bioinspired design of a CA-mimicking Phe-Zn­(II) bionanozyme amyloid-like supramolecular structures for CO_2_ conversion using catalysis approach.

## 2 Results and Discussion

### 2.1 Synthesis and Characterization of Bionanozymes

The Phe-Zn­(II) coordination complex was synthesized following a standard crystallization protocol, wherein Phe acts as a coordinating ligand to Zn^2+^ ions.^29^ Briefly, the Phe-Zn­(II) crystals were prepared using Phe and ZnCl_2_ salt (2:1 ratio) at 10 mM each in an aqueous solvent under pH 8 and heating of the solution to 60 °C. A rod-like colorless crystal was formed due to the interaction between Phe molecules and Zn­(II) ions at the air–water interface after gradual cooling to room temperature. To obtain insights into the molecular structural organization, including the supramolecular interactions of the Phe-Zn­(II) crystal, a high-quality, rod-like, colorless crystal was prepared. SC-XRD analysis showed that Phe-Zn­(II) was crystallized in the monoclinic crystal system with the noncentrosymmetric space group P2_1_ (Figures 2a, S1, and Table S1). The crystal structure of Phe-Zn­(II) revealed the formation of ((Phe)_2_Zn­(II)) complex cation in which each Zn­(II) ion is coordinated with two Phe molecules in the unit cell. The asymmetric unit confirmed the coordination of two Phe molecules to a Zn^2+^. The chemical structure of the Phe-Zn molecule was validated through its single-crystal structure (Figure 2b). Additionally, the unit cell representation showed the presence of four molecules per asymmetric unit cell arranged in a monoclinic configuration (Figure 2c). The 3D packing of the crystal structure revealed an amyloid-like β-sheet supramolecular arrangement of the Phe-Zn­(II) crystals, featuring an antiparallel alignment along the *a*, *b*, and *c* axes (Figures 2d,e, and S2). This 3D packing also demonstrated a repeating structural unit driven by intermolecular hydrogen bonding, and π–π stacking interactions within the Phe-Zn­(II) molecules (Figures 2d,e, and S2). This arrangement highlights the formation of a supramolecular cross-β-sheet, amyloid-like, hydrogen-bonded secondary structure. These superstructures extended along the crystallographic long axis, resulting in the growth of rod-like single crystals. This result clearly highlights the crucial role of stacking interactions contributed by the aromatic phenyl groups in the amyloid-like supramolecular assembly of Phe-Zn­(II). These interactions go beyond simply serving as coordination sites for Zn­(II) ions. Instead, the aromatic moieties play a pivotal role by providing an energetic contribution, structural order, and directionality to the assembly process.

**Figure 2:**
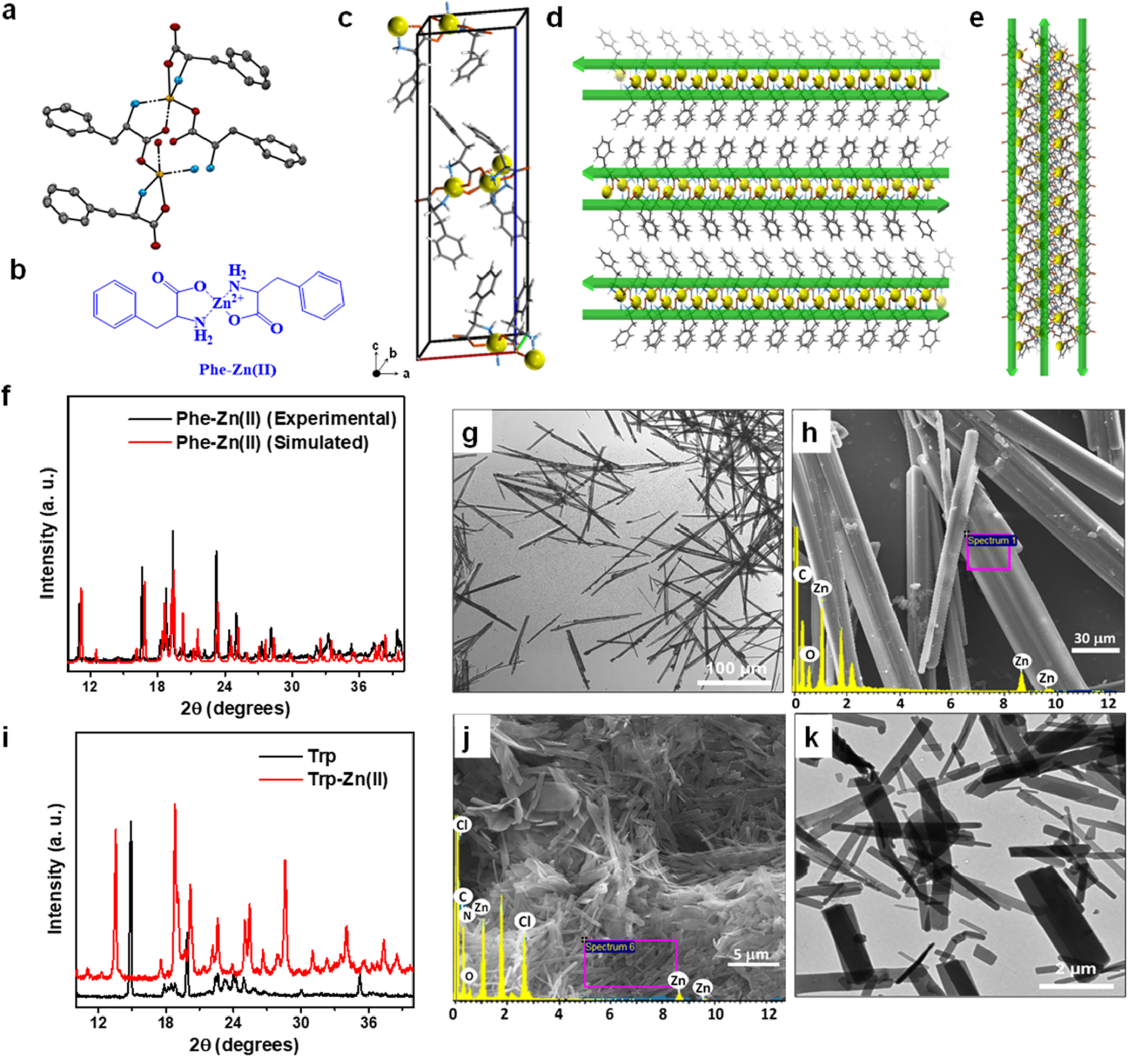
Characterization of Phe-Zn­(II) and Trp-Zn­(II) assemblies. (a) Single crystal structure of Phe-Zn­(II) crystal at a 2:1 Phe to Zn^2+^ ratio, where gray, orange, blue, yellow, and light gray colors code corresponds to carbon, oxygen, nitrogen, zinc, and hydrogen atoms, respectively, (b) Chemical structure of the Phe-Zn­(II) complex, (c) The unit cell containing 4 molecules of Phe-Zn­(II) complex in a monoclinic crystal system shown with the black letters, where *a*, *b*, and *c* are the axes and o represents the origin. (d), (e) The 3D crystal packing viewed along (d) the *a*-axis and (e) the *c*-axis showing β-sheet like supramolecular arrangement with three coordinates. Green arrows indicate the direction of Phe molecules from the N-terminus to the C-terminus. The N to C terminus-ordered Phe molecules were stabilized through hydrogen bonding and Zn­(II) ions coordination interactions. (f) Comparison of experimental and simulated PXRD pattern of the Phe-Zn­(II) crystal, exhibiting similar crystalline characteristics, peak alignment, and single-phase identification, thereby confirming the bulk-phase purity of the Phe-Zn­(II) crystals. (g) Optical microscopy, and (h) HRSEM images of the Phe-Zn­(II) crystals, confirming the rod-like supramolecular structures in a few micrometres range after the slow evaporation of the water solvent from the substrate. (i) PXRD pattern of Trp-Zn­(II) crystals, showing a different peak pattern comparison to Trp alone. (j) HRSEM, and (k) TEM images of the Trp-Zn­(II) crystals, confirming the plate-like supramolecular structures in a 2–5 μm range.

Further, the crystals were ground into a fine powder to enhance the surface area and then thoroughly analyzed. To investigate the structure further, the crystalline behavior of the Phe-Zn­(II) molecule was analyzed using PXRD, which revealed the formation of the distinct crystalline structure of the Phe-Zn­(II) crystals (Figure 2f). Notably, the PXRD pattern of the ground Phe-Zn­(II) crystals perfectly matched the simulated powder pattern derived from the SC-XRD structure, confirming the retainment of the original crystal structure in the powdered form as well (Figure 2f).

Next, microscopy techniques were utilized to explore the morphology of resultant Phe-Zn­(II) nanoassemblies. First, synthesized crystals were examined via optical microscopy, which showed the rod-shaped morphology of Phe-Zn­(II) assemblies (Figures 2g and S3a). HRSEM followed by TEM analysis showed sharp-edged crystalline assemblies (Figures 2h and S3b,c). Subsequently, we recorded EDX spectra to examine the elemental compositions (Inset Figures 2h and S4). EDX spectra of Phe-Zn­(II) assemblies clearly showed the presence of Zn metal.

Next, to increase the aromaticity and π–π stacking interactions, we synthesized Trp-Zn­(II) crystals in which two tryptophan molecules were coordinated with Zn^2+^ ions. To prepare the Trp-Zn­(II) crystals, Trp was dissolved in distilled water at pH 8, resulting in a clear solution. The clear Trp solution was then slowly added to the separately prepared ZnCl_2_ solution, leading to the immediate formation of a curdy precipitate. After shaking the mixture for several minutes, a clear solution was obtained. Trp-Zn­(II) crystals formed at the bottom of the solution after several hours. The synthesized compound was employed for PXRD analysis. PXRD spectra revealed the formation of a distinct crystalline structure compared to the amino acid Trp (Figure 2i). The PXRD analysis also confirmed the monoclinic arrangement of the Trp-Zn­(II) crystal, with cell dimensions of *a* = 41.625(19) Å, *b* = 5.483(3) Å, *c* = 9.365(4) Å, along with the angle β = 109.300(19)° (Table S2). Next, the surface morphology of the Trp-Zn­(II) microcrystals was analyzed using HRSEM, showing the presence of aggregates composed of a large number of sheet-like structures 5 μm in length (Figure 2j). Furthermore, EDX analysis revealed the presence of Zn metal (Inset Figures 2j, and S5). Next, TEM analysis of Trp-Zn­(II) showed the presence of sharp-edged 2D microcrystalline assemblies (Figure 2k). Electron microscopy showed that the crystal size for the phenylalanine was more monodispersed compared to the tryptophan.

Furthermore, in order to learn about the molecular structures of both complexes, namely Phe-Zn­(II), and Trp-Zn­(II), we performed electrospray ionization mass spectrometry (ESI-MS) analysis in water as solvent. We observed the *m*/*z* peaks at 393.4 (Figures 3a, and S6) and 471.5 (Figure S7) in positive ion mode for ((Phe)_2_Zn)^2+^, and ((Trp)_2_Zn)^2+^ complex cations in the water solvent, which further supported the XRD data (Figure 2). Moreover, the ESI-MS analysis confirmed the presence of both complexes in water. XPS analysis of the Phe-Zn­(II) crystals was conducted to investigate the nature of chemical bonding on the surface. The XPS survey spectrum confirmed the presence of Zn, along with C, N, and O (Figure 3b). In the high-resolution Zn 2p region, the observed peaks were symmetrical, with binding energies of 1021.10 and 1044.09 eV, corresponding to the Zn 2p_3/2_, and Zn 2p_1/2_ transitions, respectively (Figure 3c). The energy separation between these peaks is 22.99 eV, which aligns well with the reported values for metallic zinc. Notably, no evidence of zinc oxide was detected, as the Zn 2p_3/2_, and Zn 2p_1/2_ peaks characteristic of ZnO (typically observed at 1021.7 and 1044.8 eV, respectively) were absent in the Phe-Zn­(II) XPS spectrum.^54^ XPS C 1s spectra revealed peaks corresponding to various functional groups present in the Phe-Zn­(II) (Figure 3d). Peaks observed at binding energies of 284.6, 285.1, 286.1, and 288.6 eV confirmed the presence of C=C, C–N, C–O, and O–C=O interactions, respectively. Furthermore, the N 1s spectrum displayed a peak at 399.6 eV, indicative of the C-NH bond (Figure 3e). The O 1s region exhibited a single, low-intensity peak at 531.2 eV, suggesting the presence of Carbonyl functional group (Figure 3f). Results of XPS suggests proper binding of Phe with Zn successfully and the formation of the Phe-Zn­(II) assemblies.

**Figure 3:**
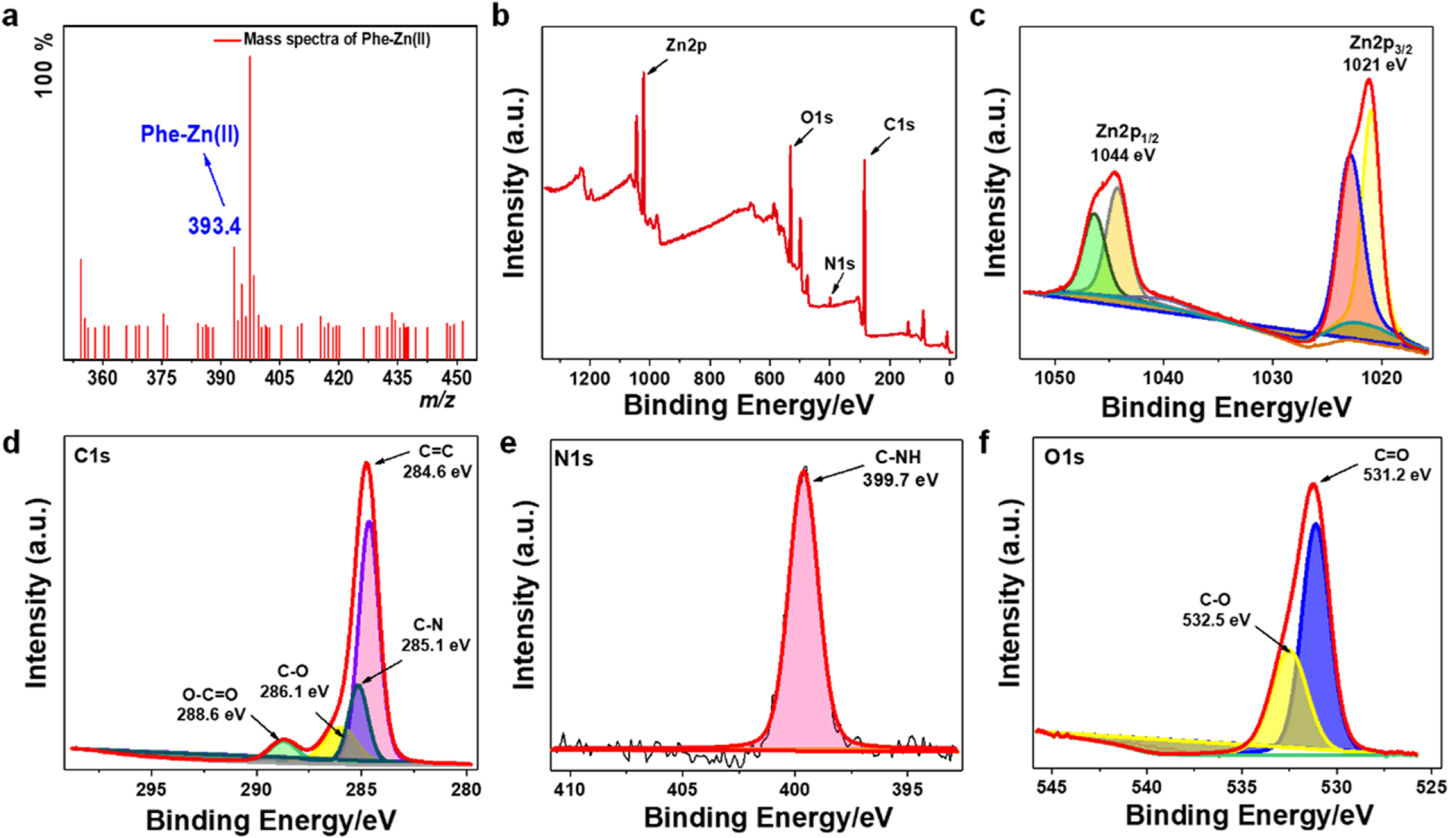
XPS characterization of Phe-Zn­(II) assemblies. (a) ESI-MS spectra of Phe-Zn­(II). (b) XPS survey spectra showing the presence of Zn along with C, N and O with their respective spin states. (c) Peaks corresponding to binding energies 1021 and 1044 eV confirm the presence of Zn^2+^ in spin states like Zn 2p_3/2_ and Zn 2p_1/2_. (d) C 1s XPS spectra with peaks at 285.1, 284.6, 286.1, and 288.6 eV, corresponding to C–N, C=C, C–O and O–C=O functional moieties, respectively, in the Phe-Zn­(II) molecules. (e) N 1s XPS spectra showing a peak at 399.6 eV corresponding to a C-NH bond in the Phe-Zn­(II) molecules. (f) O 1s XPS spectra showing peaks at 532.5 and 531.2 eV, corresponding to C–O and C=O bonds, respectively, in the Phe-Zn­(II) molecules.

To further comprehensively elucidate the surface chemical composition and electronic environment of the synthesized Trp–Zn­(II) nanomaterial, X-ray photoelectron spectroscopy (XPS) was employed. The wide-scan XPS survey spectrum provided a robust confirmation of the elemental constituents present on the material’s surface, clearly indicating the presence of zinc (Zn), oxygen (O), nitrogen (N), and carbon (C). These elements were identified based on their characteristic binding energy peaks observed at 1022.7 eV (Zn 2p), 529.6 eV (O 1s), 398.5 eV (N 1s), and 282.1 eV (C 1s), respectively (Figure S8). Further insights into the chemical states and bonding environments of these elements were obtained through high-resolution spectral deconvolution. Specifically, the Zn 2p region revealed two distinct peaks centered at 1023.2 and 1046.5 eV, which correspond to Zn 2p_3/2_ and Zn 2p_1/2_, respectively (Figure 4a). These values are consistent with the +2 oxidation state of zinc (Zn^2+^), thus confirming the presence of divalent zinc species in the Trp–Zn structure. A detailed analysis of the high-resolution C 1s spectrum uncovered multiple components, indicating the presence of diverse carbon-based functional groups. Peaks were observed at binding energies of 284.5 eV (C=C, sp^2^-hybridized carbon), 285.1 eV (C–N), and 288.6 eV (O–C=O), suggesting the successful incorporation of oxygen- and nitrogen-containing functionalities within the material (Figure 4b). In addition, the high-resolution N 1s and O 1s spectra further supported the structural complexity and surface modification of the Trp–Zn­(II) assemblies (Figures 4c and d). The N 1s peak at 400.1 eV was attributed to the presence of C–NH moieties, indicative of amide or carbamate-type functionalities. Similarly, the O 1s peak at 532.3 eV is characteristic of oxygen atoms in carbonyl (C=O) functional group forms. Collectively, the XPS findings not only confirm the successful integration of Zn^2+^ ions and functional organic moieties but also substantiate the deliberate chemical functionalization of the Trp–Zn­(II) nanomaterial. These results underscore the material’s well-defined surface chemistry and structural integrity, which are crucial for its anticipated applications in catalysis, sensing, or biomedical domains.

**Figure 4:**
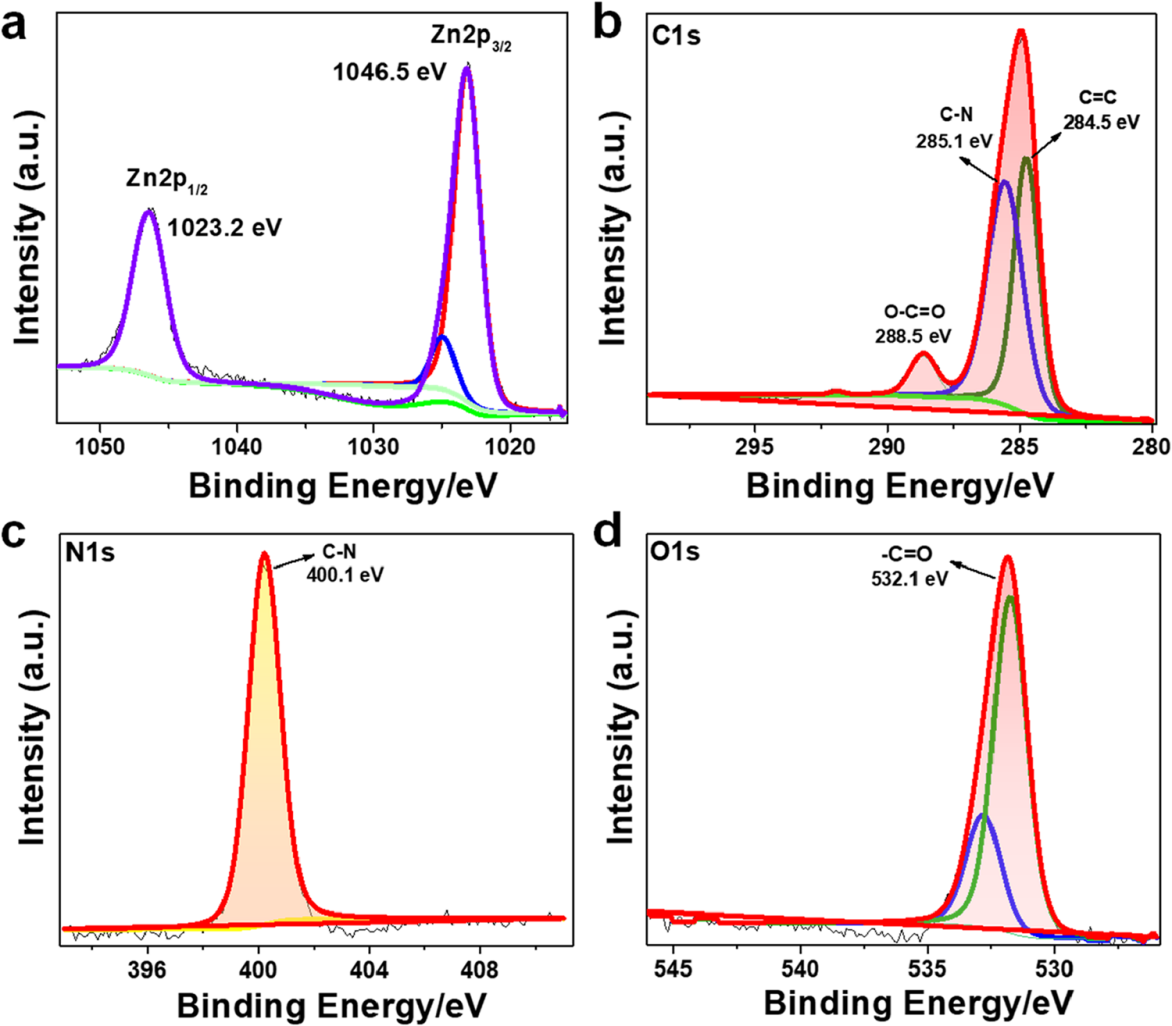
XPS characterization of Trp-Zn­(II) assemblies. (a) Peaks corresponding to binding energies 1023.2 and 1046.5 eV confirm the presence of Zn^2+^ in spin states like Zn 2p_3/2_ and Zn 2p_1/2_. (b) C 1s XPS spectra with peaks at 285.1, 284.5, and 288.5 eV, corresponding to C–N, C=C, and O–C=O bonds, respectively, in the Trp-Zn­(II) molecules. (c) N 1s XPS spectra showing a peak at 400.1 eV corresponding to a C-NH bond in the Trp-Zn­(II) molecules. (d) O 1s XPS spectra showing peaks at 532.1, corresponding to C=O bonds, respectively, in the Trp-Zn­(II) molecules.

### 2.2 CO_2_ Conversion and Quantification Using Phe-Zn­(II) and Trp-Zn­(II) Bionanozymes

According to recent reports, when CA is embedded into a microporous zeolite imidazolate framework via physical absorption, it can show efficient CO_2_ conversion and catalysis.^38^ This approach offers a new and alternative route to design novel candidates for CO_2_ conversion, yet the use of expensive natural enzymes and the critical embedded process are the main challenges. To overcome the aforementioned challenge, this work offers an alternative approach by using the designed Phe-Zn­(II) amyloid-inspired nanoassemblies for an efficient CO_2_ conversion process. CO_2_ catalysis experiment using the Phe-Zn­(II), and Trp-Zn­(II) crystals was performed by placing the crystals in an aqueous reactor continuously supplemented with 5 vol % CO_2_/He, and analyzing the amount of CO_2_ consumed using in-line gas chromatography (GC) (Figure S9). The outlet CO_2_ concentration was measured using a gas chromatography equipped with a thermal conductivity detector (TCD). The gas flow was set to a total of 100 standard cm^3^/min. Initially, the empty reaction chamber was purged with 5 vol % CO_2_/He, and GC was used to confirm both that the CO_2_ composition in the feed stream was 5 vol % and that no residual N_2_/O_2_ was detected inside the reactor. When a steady CO_2_ concentration was measured by the GC, 80 mL of deionized distilled water (DDW) was added to the reaction vessel using a syringe through a hermetic seal (so as not to introduce air), and the water was purged with 5 vol % CO_2_/He for 30 min to achieve saturation of CO_2_ in the water. The CO_2_-saturated water in the reactor ensures that any decrease in CO_2_ concentration measured in the outlet of the reactor after the introduction of the catalyst is not caused by its absorption through the water. The steady-state decrease in the vol % of CO_2_ as measured by the GC after adding the water was due to the presence of water vapor in the exit stream which is carried by the otherwise dry 5% CO_2_/He, hence slightly lowering the volume fraction of CO_2_ in the sample. Once a steady CO_2_ vol % was achieved in the CO_2_-saturated water, 20 mL of a freshly prepared solution of each crystal (850 mg/35 mL) was injected into the reaction vessel using a syringe through the hermetic seal while continuously flowing 5% CO_2_/He. GC measurements were taken starting 5 min after the catalyst addition to the reactor until a steady-state was reached (Figure 5). Phe-Zn­(II) showed CO_2_ conversion of ∼18% (Figure 5a), whereas the Trp-Zn­(II) crystal showed ∼14.5% CO_2_ conversion under the same conditions (Figure 5a). Moreover, 91 and 73 mmol/min of CO_2_ were consumed per gram of Phe-Zn­(II) and Trp-Zn­(II) catalysts, respectively. Furthermore, PXRD analysis was performed to test the structural stability of the crystals after catalysis. No significant crystallographic changes were observed in the PXRD pattern of both Phe-Zn­(II) and Trp-Zn­(II) crystals following the catalytic process (Figure 5b,5c). To further evaluate structural robustness and practical applicability, recyclability experiments were performed over five consecutive catalytic cycles. After each cycle, the Phe-Zn­(II) bionanozyme was recovered by centrifugation, thoroughly washed with double-distilled water, and reused without additional treatment. The catalyst retained more than 96% of its initial catalytic activity after five cycles (Figure S10), and PXRD patterns confirmed preservation of crystallinity. These results demonstrate both catalytic durability and structural stability. To contextualize the catalytic performance of Phe-Zn­(II), we compared it with representative carbonic anhydrase mimics reported in the literature, including Zn-amino acid complexes, peptide-derived supramolecular assemblies, immobilized enzyme systems, and Zn single-atom nanozymes. Under comparable aqueous and near-neutral conditions, the CO_2_ conversion efficiency of Phe-Zn­(II) (∼18%) falls within the upper range of small-molecule CA mimics, while offering significantly reduced structural complexity and improved postreaction crystallographic retention. A comparative summary is provided in Table S4 (Supporting Information), highlighting reaction conditions, structural characteristics, and catalytic performance metrics. Unlike immobilized CA systems, which may suffer from enzyme denaturation or leaching, the Phe-Zn­(II) framework preserves its crystalline architecture after catalysis, underscoring its robustness. Thus, the designed CA enzyme mimicking Phe-Zn­(II) bionanozyme exhibited high catalytic CO_2_ conversion activity, along with ease of synthesis, low-cost, high catalytic activity, and structural stability.

**Figure 5:**
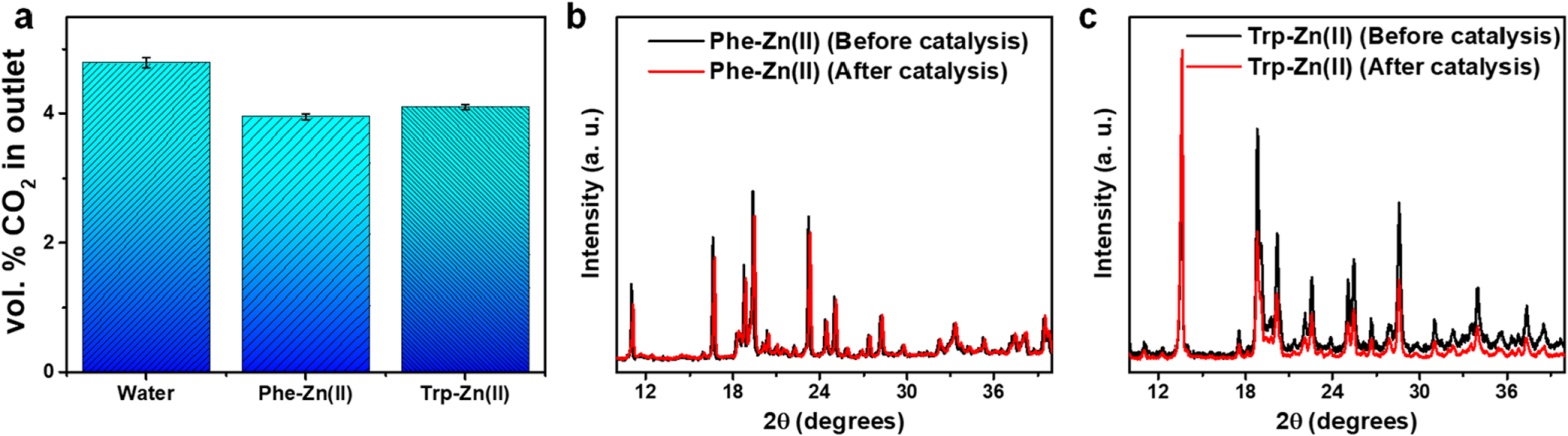
Catalytic CO_2_ conversion activity of bionanozymes. (a) CO_2_ conversion using Phe-Zn­(II) and Trp-Zn­(II) crystals in water. (b) PXRD pattern of Phe-Zn­(II) crystals before and after catalysis. (c) PXRD pattern of Trp-Zn­(II) crystals before and after catalysis.

### 2.3 Molecular Reaction Mechanisms for CO_2_ Conversion Using Computational Models

To better understand how Phe-Zn­(II) mimics CA in catalyzing CO_2_ hydration, we used *ab initio* calculations to analyze the step-by-step reaction pathway. Starting from the crystal structure, we designed a catalytic center (**Model I**) consisting of two Phe ligands, a Zn^2+^ ion, a CO_2_ molecule, and water molecules. Since the catalyst is a solid with relatively small pores and insoluble in water, we focused on the reaction mechanism of catalytic conversion of CO_2_ on the catalyst surface. As a result, we included environmental water molecules only on the side of the zinc-coordinated water molecule. The reaction occurs in three steps (Figure 6): OH^–^ generation, nucleophilic attack, and carbonic acid formation. In the first step, Zn^2+^ activates its bound water molecule, and the latter transfers a proton to the amino or carboxyl group. We studied three possible pathways. One pathway featured proton transfer to the amine group (black line), forming a hydroxide ion with a free energy barrier height of 4.5 kcal/mol (**TS2/3**). Then, upon the approach of CO_2_ to the catalytic site, the hydroxide ion nucleophile attacked the carbon atom of CO_2_, resulting in the formation of a new C–O bond (from 2.54 Å in compound **4** to 1.40 Å in compound **5**) through the transition state **TS4/5**. This process, the rate-limiting step, involves a 12.3 kcal/mol free energy barrier, which is sufficiently low to enable the spontaneous occurrence of the reaction at room temperature. The outcome of this step was the formation of a bicarbonate ion, which may either remain in solution or proceed to form carbonic acid. In the carbonic acid formation step, the bicarbonate ion, coordinated with the zinc ion in intermediate **5**, accepted a proton from the positively charged amine group, accomplishing the formation of carbonic acid with a free energy barrier height of 3.3 kcal/mol. Ultimately, the product dissociated from the active site, and a fresh water molecule entered (complex **8**), with a free energy change of 4.4 kcal/mol.

**Figure 6:**
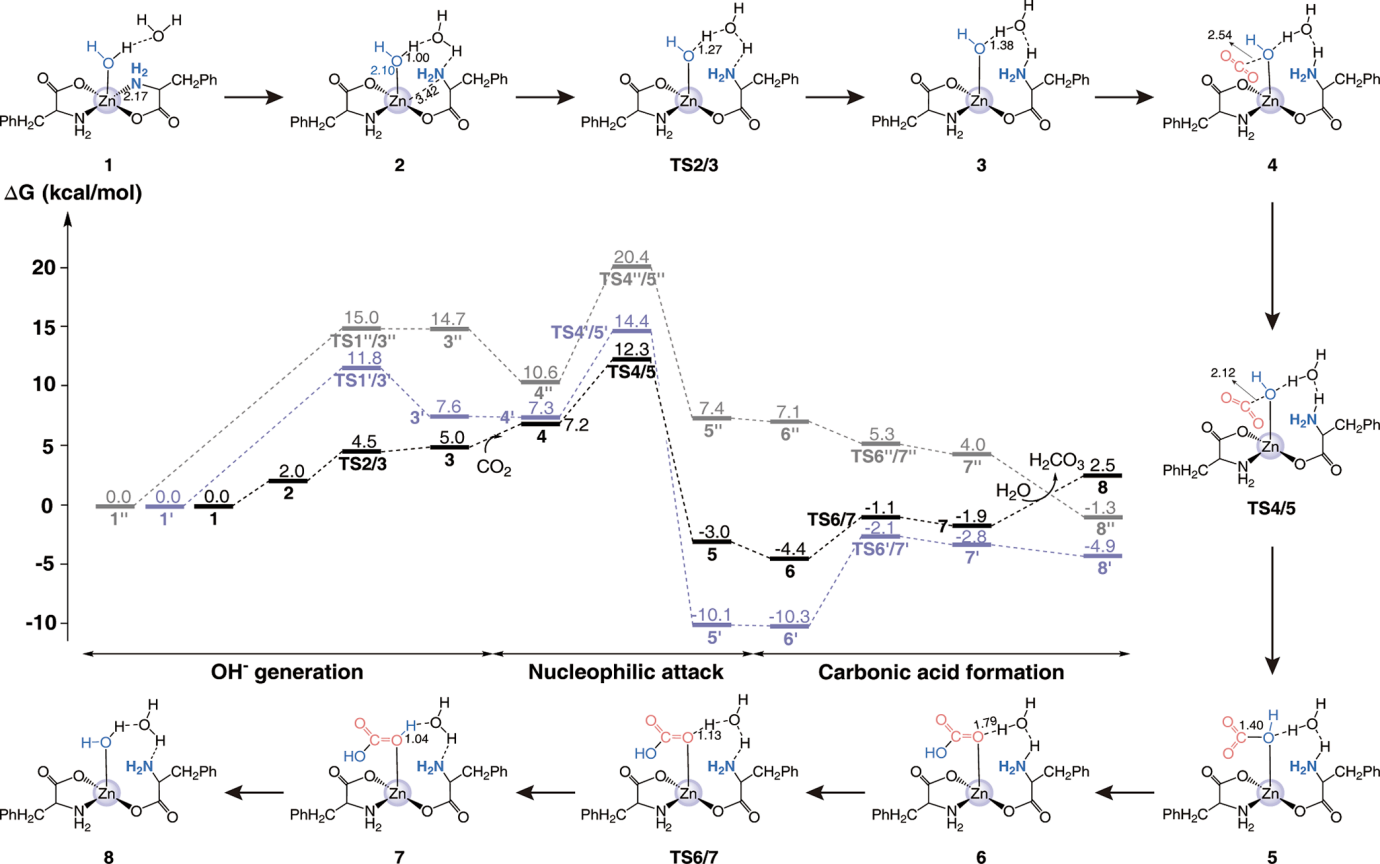
Molecular reaction mechanisms for catalytic CO_2_ conversion in **Model I**. Three distinct reaction pathways are investigated, differing primarily in the activation mechanism for the zinc-bound water molecule, with distances labeled in angstroms (Å). One pathway features proton transfer to the amine group (black line). A second pathway involves proton transfer to the carboxyl group, mediated by an unimolecular water bridge (gray line). The third pathway is the proton transfer to the carboxyl group through a bimolecular water bridge (purple line).

The other two paths involved proton transfer from the zinc-bound water to the carboxyl group, mediated by an unimolecular or a bimolecular water bridge (Figure 6). The reaction mechanisms of these two reaction paths resembled those of the first reaction path. Although the rate-limiting steps of these pathways feature closely related free energy barriers (approximately 2.1 kcal/mol apart), computational results suggest that the deprotonation of the zinc-bound water followed by proton transfer to a neighboring amine group is the most probable mechanism. Nevertheless, considering the precision of DFT calculations, the possibility that nearby carboxyl groups assist in proton transfer via two water molecules acting as a bridge, forming the active site during CO_2_ hydration, cannot be entirely dismissed. Overall, the activation energy barrier was only 12.3 kcal/mol, and the energies of products **5** and **7** were significantly lower than that of reactant **4**, confirming that the reaction can proceed rapidly at room temperature.

To investigate the role of the catalytic center, we further generated a simplified model, **Model II**, by replacing the benzene ring with a methyl group. Calculations yielded a consistent conclusion (Figure 7): the nucleophilic attack is the rate-limiting step, and both carboxyl and amino groups can act as proton acceptors. **Models I** and **II** share a similar reaction mechanism, which supports the validity of the proposed mechanism. However, methyl substitution on the phenyl ring increases the energy barrier of the rate-determining step, highlighting the influence of side-chain modifications on catalytic efficiency. The aromatic side chain, due to its stronger electron-withdrawing effect, enhances the Lewis acidity of the Zn­(II) center, thereby facilitating activation and lowering the energy barrier for catalysis.

**Figure 7:**
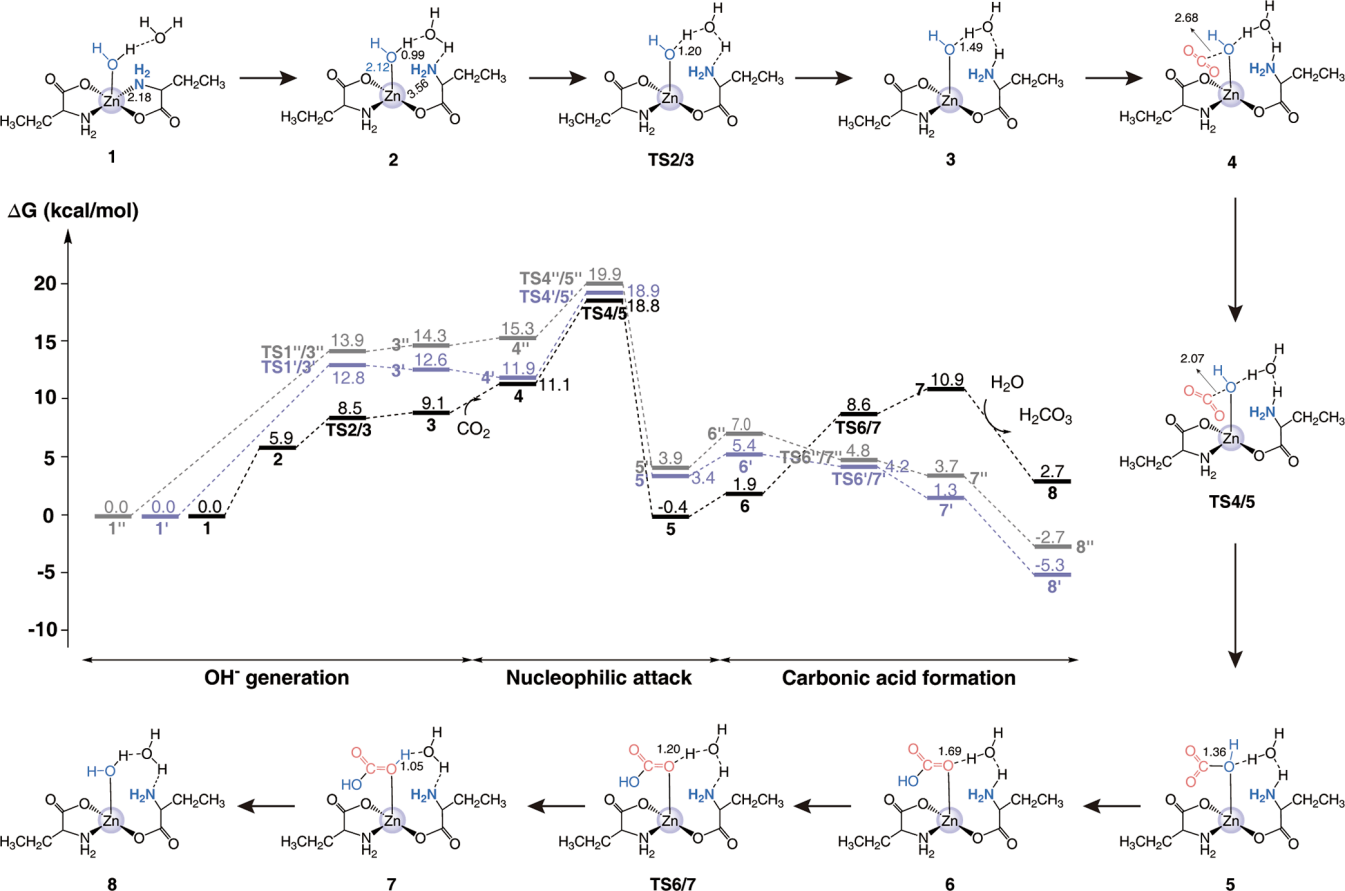
Molecular reaction mechanisms for catalytic CO_2_ conversion in **Model II**. Three distinct reaction pathways are investigated, differing primarily in the activation mechanism for the zinc-bound water molecule, with distances labeled in angstroms (Å). One pathway features proton transfer to the amine group (black line). A second pathway involves proton transfer to the carboxyl group, mediated by an unimolecular water bridge (gray line). The third pathway is the proton transfer to the carboxyl group through a bimolecular water bridge (purple line).

To further explore the role of coordinating amino acids, we replaced the phenyl ring of Phe with an indole ring in **Model I** to construct the Trp-Zn­(II) monomer, **Model III**, and calculated the energy barrier of the nucleophilic attack step along the most favorable reaction pathway. As shown in Table S3, the energy barrier of **Model III** was 3.1 kcal/mol higher than that of **Model I**, consistent with the experimental observation that the CO_2_ conversion of Trp-Zn­(II) decreased by 3.5% compared to that of Phe-Zn­(II). In contrast to indole, the phenyl ring exhibits a larger electron-withdrawing effect, which intensifies the Lewis acidity of Zn­(II), accelerates Zn–OH_2_ bond activation, and decreases the energy barrier associated with the nucleophilic attack step. Additionally, structural differences in the crystal forms of different amino acids may also influence the catalytic activity. The consistency in the observed trends by both methods supports the validity of the computational model.

Previous studies based on quantum mechanical calculations have reported that the rate-determining step of the catalytic cycle of CA is associated with proton transfer, with a free energy barrier height of 10.0–14.6 kcal/mol.^55,56^ This value is comparable to the catalytic barrier calculated in this work for the Phe-Zn­(II) system, indicating the potential efficiency of Phe-Zn­(II) for CO_2_ conversion. Notably, the two systems exhibit distinct rate-determining steps, showing the importance of the local microenvironment in modulating the catalytic mechanism and efficiency of CO_2_ conversion: in the Phe-Zn­(II) system, the amino group can act as a stable proton acceptor, facilitating efficient proton transfer. In contrast, in the CA active site, the additional proton is often positioned near hydrophobic residues or acidic amino acids such as Asp and Glu, which may hinder the stabilization of the transition state and increase the barrier for proton transfer.

The amyloid-like cross-β-sheet supramolecular architecture of Phe-Zn­(II) indeed creates a distinct microenvironment compared to the active site of natural CA. Unlike the active site of natural CA, which features a specific substrate-binding pocket and neighboring amino acid residues that are intricately involved in catalysis, the Phe-Zn­(II) assembly provides a confined space. This behavior in Phe-Zn­(II) may be attributed to the hydrophobic phenyl groups, which enhance the local CO_2_ concentration near the catalytic Zn center, while interfacial water molecules within the assembly may facilitate proton shuttling. This organized, yet fundamentally different, microenvironment in Phe-Zn­(II) likely contributes to the observed difference in the rate-determining step compared to natural CA, where a histidine-mediated proton relay network is dominant and precisely positioned within a protein scaffold optimized for catalysis.

## 3 Conclusions

This study presents the development of a highly efficient Phe-Zn­(II) bionanozyme designed to mimic the CO_2_ sequestration activity of CA. The study was conducted in an aqueous solution (pH 7.0), at a temperature of 25 °C, and at ambient pressure. These conditions were used for catalytic evaluation and stability assessment. The investigation of the single-crystal structure of Phe-Zn­(II) revealed an amyloid-like supramolecular cross β-sheet framework, which is integral to its functionality. Remarkably, the Phe-Zn­(II) bionanozyme achieved a CO_2_ conversion rate of ∼18% from an aqueous solution, thereby demonstrating superior performance compared to the Trp-Zn­(II) variant, which converted ∼14.5% under the same experimental conditions. Further analysis using PXRD indicated no significant changes in the crystalline properties of the Phe-Zn­(II) bionanozyme throughout the catalytic process. This stability underscores the bionanozyme’s exceptional robustness and potential for practical applications in CO_2_-sequestering technologies. Furthermore, we constructed the Phe-Zn­(II) monomer model (**Model I**) to explore the reaction pathway and found that proton transfer to the amine is most favorable, with the nucleophilic attack as the rate-determining step, having a barrier of 12.3 kcal/mol, making the reaction suitable for room temperature. To investigate the source of Phe-Zn­(II)’s high catalytic activity, we replaced the phenyl group with indole to create **Model III** and then calculated their rate-determining barriers. The results show that **Model II** has the lowest barrier, which matches its highest catalytic activity, mainly due to the electron-withdrawing effect of the phenyl group, enhancing Zn^2+^’s Lewis’s acidity.

Notably, Phe-Zn­(II) emerged as a low-cost, sustainable, and high-performance sorbent for carbon conversion, offering significant promise for advancing renewable energy and environmental sustainability through the development of durable and efficient catalysts. Remarkably, Phe-Zn­(II) shows several advantages compared to other artificial hydrolases including low molecular mass, high catalytic efficiency, and structural stability.^29^ This research highlights the potential of metal-coordinated amino acid crystals as durable, sustainable catalysts for CO_2_ conversion, with significant implications for renewable energy and environmental sustainability. Continued research seeks to deepen our understanding and broaden the use of this ground-breaking bionanozyme, paving the way for notable progress in biomimetic enzymology, environmental conservation, and biotechnology.

## 4 Experimental Section

### 4.1 Materials

All the chemicals, including amino acids, were purchased from Sigma-Aldrich (purity >98%). All materials were utilized in their original state without additional purification. Water was processed using a Millipore purification system (Biological Industries, Beit Haemek, Israel) with a minimum resistivity of 18.2 MΩ cm.

### 4.2 Fabrication of Phe-Zn­(II) Crystal

The Phe-Zn­(II) crystals were synthesized by reacting 2 equiv of Phe (10 mM), 2 equiv of NaOH (10 mM), and 1 equiv of ZnCl_2_ (10 mM) in water at 60 °C for 15 min rod-like, colorless crystals formed during the gradual cooling stage. The crystals were filtered using Whatman filter paper to remove any remaining contaminants which were not reacted, washed with deionized water followed by ethanol 3 times, dried under vacuum, and subsequently used for further analysis.

### 4.3 Fabrication of Trp-Zn­(II) Crystal

In order to prepare Trp-Zn­(II) crystals, 50 mM Trp was dissolved in 40 mL of distilled water. A 50 mM NaOH solution was slowly added to dissolve the Trp and obtain a clear solution. The clear solution was slowly added to 50 mM of ZnCl_2_ in 20 mL of distilled water, and the formation of a curdy precipitate took place immediately. After shaking well for several minutes, a clear solution was obtained and transferred into a 500 mL beaker. The Trp-Zn­(II) crystal formation takes place at the bottom after 4–6 h. To remove any unreacted contaminants, the crystals were washed 3 times with distilled water and filtered, dried under high vacuum, and subsequently used for further analysis.

### 4.4 Crystal Preparation and Data Collection

Phe-Zn­(II) crystals used for data collection were prepared using Phe and ZnCl_2_ salt (2:1 ratio) in water at pH 8 and heating at 60 °C. Rod-like crystal formation took place at the water–air interface after several minutes of gradual cooling. For data collection, the crystals were coated with Paratone oil (Hampton Research), mounted on a MiTeGen cryo-loop, and flash-frozen in liquid nitrogen. Single-crystal diffraction data were collected at 100 K using a Rigaku XtaLAB Synergy R rotating anode system equipped with a HyPix-Arc 150 detector and Cu Kα radiation (λ = 1.54184 Å).

### 4.5 Processing and Structural Refinement of Crystal Data

Diffraction data were collected and processed using the CrysAlisPro 1.171.41.111a software (Rigaku OD, 2021). The structures were solved using direct methods with SHELXT-2018 and refined by full-matrix least-squares on F^2^ using SHELXL-2016. All atoms, except for hydrogen, were refined independently and anisotropically. Hydrogens were placed in calculated positions and refined using a riding model. Details of crystal data collection and refinement parameters are provided in Supporting Table S1, with complete data available in the CIF file included as Supporting Information. The crystallographic data for the Phe-Zn­(II) crystal have been deposited in the CCDC under accession number 2412875.

### 4.6 Powder X-ray Diffraction (PXRD)

The Phe-Zn­(II) and Trp-Zn­(II) crystals were prepared in water, filtered, and thoroughly dried under vacuum. The powdered crystals were placed on a quartz zero-background sample holder. PXRD patterns were obtained using a Bruker D8 Discover diffractometer (Bruker, Germany) equipped with Göbel mirrors for beam parallelization and a LYNXEYE-XE linear detector. Data collection was performed at room temperature over a 2θ scan range of 5–80°. The background was eliminated to focus solely on the diffraction peaks.

### 4.7 Optical Microscopy

The Phe-Zn­(II), and Trp-Zn­(II) crystals were prepared in water, drop-cast onto a glass slide, and directly observed using a Nikon Eclipse Ti-E fluorescence microscope under bright-field channels.

### 4.8 High-Resolution Scanning Electron Microscopy (HR-SEM)

A 10 μL aliquot of the crystals dispersed in water was allowed to dry on a silicon wafer under ambient conditions overnight and coated with Au. HR-SEM images were acquired using a ThermoFisher, Quanta 200 FEG ESEM operated at an accelerating voltage of 5 kV. Energy-dispersive X-ray spectroscopy (EDX) analysis of Phe-Zn­(II) and Trp-Zn­(II) crystals was also performed using the same instrument under the same conditions operated at an accelerating voltage of 20 kV. Three measurements were conducted and the results were averaged to ensure accuracy.

### 4.9 Transmission Electron Microscopy (TEM)

A 10 μL aliquot of the Phe-Zn­(II) and Trp-Zn­(II) crystals dispersed in water was drop-cast onto a 400-mesh carbon-stabilized Formvar-coated Cu grid (Ted Pella, California, USA). The sample was allowed to adhere to the grid surface for 5 min, after which excess solution was removed using a lint-free tissue. The grid was dried at room temperature before imaging. Sample morphology was examined using a JEM-1400 TEM (JEOL, Tokyo, Japan) operated at an accelerating voltage of 80 kV.

### 4.10 Mass Spectrometry

Mass spectrometry analysis of the Phe-Zn­(II) and Trp-Zn­(II) complexes was performed using an Acquity UPLC system coupled with a TQD XEVO triple quadrupole mass spectrometer equipped with an electrospray ionization (ESI) source (Waters, Milford, MA, USA). Measurements were conducted in the positive ionization modes via ESI.

### 4.11 Spectroscopic Data

#### 4.11.1 Complex 1 [Phe-Zn­(II), (M)]

ESI Mass spectra (ESI^+^), *m*/*z* calculated for (M+H)^+^ C_18_H_21_N_2_O_4_Zn: 393.1, found: 393.4.

#### 4.11.2 Complex 2 [Trp-Zn­(II), (M)]

ESI Mass spectra (ESI^+^), *m*/*z* calculated for (M+H)^+^ C_22_H_23_N_4_O_4_Zn: 471.1, found: 471.5.

### 4.12 X-ray Photoelectron Spectroscopy (XPS)

The dispersed Phe-Zn­(II) crystals were drop-cast on the silicon wafer, and dried. XPS spectra were collected via ESCALAB QXi X-ray Photoelectron Spectrometer Microprobe (Thermo Fisher Scientific, USA), using XPS (650 μm) and dual beam method.

### 4.13 Gas Chromatography (GC)

GC analysis was performed using an SRI (California, USA) gas chromatograph fitted with a flame-ionization detector (FID) and thermal conductivity detector (TCD) (Figure S9). Both detectors were exposed to the sample using a helium carrier. The injector consisted of a constant-volume 1 mL injector loop connected to a 10-port valve, allowing for the injection of exiting gases from the reactor for analysis and for continuous in-line flow. A 6′ packed Hayesep 5A column was used to separate the reaction gases. The injector, column, and TCD detector were all kept at 110 °C in order to prevent condensation of moisture carried by the exiting gases from the reactor. The FID was operated at 300 °C with a H_2_/Air feed. A calibration curve of various concentrations of CO_2_ in He was used in order to quantify the amount of CO_2_ in the exit stream of the reactor based on the peak area. The detector used was TCD. The GC is equipped with both a thermal conductivity detector (TCD) and a methanizer followed by a flame ionization detector (FID). The FID can only detect C–H bonds, so the methanizer converts the CO_2_ to CH_4_, which is subsequently detected by the FID and used to quantify the amount of CO_2_. The FID was used to quantify the CO_2_, while the TCD was used to make sure that there was no air (N_2_/O_2_) in the reactor. The presence of air in the reactor would have impacted the fraction of CO_2_ which was measured, and complicated our analysis.

### 4.14 Computational Analysis

Cluster models were built for the *ab initio* calculations. **Model I** consisted of two Phe residues coordinated with Zn^2+^ through their oxygen and nitrogen atoms. A few water molecules nearby the active site were also added to mimic the solvation effect at the interface. The carbon atoms of the phenyl ring in the Phe residues were fixed to mimic the constraints imposed by π-π stacking interactions. The initial geometry was built using the structure derived from the crystal structure (CCDC ID: 1850564). To elucidate the influence of side-chain electronic effects on catalytic activity, **Models II** and **III** were derived from **Model I** by substituting the phenyl group with methyl and indole groups, respectively. The substituents were positionally constrained to reflect the π-stacking observed in the crystal structure, with the rest of the coordination framework preserved.

All geometry optimizations and frequency analyses were performed using the M06–2X/^57^ 6–31G*^58^ method with the SMD solvation model^59^ and Zn^2+^ ions were described using LANL2DZ.^60^ For electronic energy calculations, the larger cc-pVTZ^61^ basis set was used. Thermodynamic corrections from the frequency analysis were applied to the solution-phase single-point energies to obtain Gibbs free energy, with an additional correction of 1.9 kcal/mol,^62,63^ for the solution-phase Gibbs free energies used in mechanistic discussions. All *ab initio* calculations were performed using the Gaussian16 program version Rev. A.03.^64^

## Supplementary Material



## Notes

ORTEP diagram and 3D crystal packing from SC-XRD of Phe-Zn­(II) crystal; optical microscopy, HRSEM, TEM, and EDX analysis of Phe-Zn­(II) crystal; EDX analysis of Trp-Zn­(II) crystal; ESI mass spectra of Phe-Zn­(II) and Trp-Zn­(II) complexes; XPS survey spectra of Phe-Zn­(II); picture of GC instrument set up for the experiment of CO_2_ catalysis; recyclability of Phe-Zn­(II); experimental details for single crystal and references (Figure S1–S10)**;** crystallographic data collection and refinement statistics data of Phe-Zn­(II) crystal from single crystal structure; cell parameter of Trp-Zn­(II) crystals; effect of substituents on Zn^2+^, Zn^2+^–OH_2_ bonding, and rate-determining barrier in intermediate 2; and comparison of CO_2_ hydration performance of Phe-Zn­(II) with reported CA mimics (Table S1–S4) (PDF)
