# Supplementary material for: CO2 Conversion by a Metal-Coordinated Single Amino Acid Carbonic Anhydrase Enzyme Mimic
Source: ACS Appl Mater Interfaces. 2026 Mar 26;18(13):18884–97. doi: 10.1021/acsami.5c26143 (PMC13067265; doi:10.1021/acsami.5c26143)
Supplement: Supplementary file 1 [file am5c26143_si_002.pdf]

Supporting Information

CO_2 Conversion by a Metal-Coordinated Single Amino Acid Carbonic Anhydrase Enzyme Mimic

Adithya Ramesh[a#], Xiaoyu Wang[b#], Subrat Vishwakarma[c], Oren Ben-Zvi[d], Linda J. W. Shimon[e], Sigal Rencus-Lazar[f], Pandeeswar Makam[c], Hao Dong[b]*, Ehud Gazit[f]*, Om Shanker Tiwari[a,f]* and Brian A. Rosen[g]*

[a] Department of Chemistry, National Institute of Technology Warangal, Hanamkonda-506004, Telangana, India.
[b] State Key Laboratory of Analytical Chemistry for Life Science, Kuang Yaming Honors School, Chemistry and Biomedicine Innovation Center (ChemBIC), Institute for Brain Sciences, Nanjing University, Nanjing 210023, China.
[c] Department of Chemistry, Indian Institute of Technology (BHU), Varanasi, UP, 221005, India.
[d] School of Plant Sciences and Food Security, The George S. Wise Faculty of Life Sciences, Tel Aviv University, Tel Aviv, Israel.
[e] Department of Chemical Research Support, Weizmann Institute of Science, Rehovot 7610001, Israel.
[f] The Shmunis School of Biomedicine and Cancer Research, The George S. Wise Faculty of Life Sciences, Tel Aviv University, Tel Aviv 6997801, Israel.
[g] Department of Materials Science and Engineering, Tel Aviv University, Tel Aviv 6997801, Israel.

Author Information
#Equal contribution
*Corresponding Authors
Prof. Ehud Gazit
Address: The Shmunis School of Biomedicine and Cancer Research, The George S. Wise Faculty of Life Sciences, Tel Aviv University, Tel Aviv 6997801, Israel.
Email: ehudg@post.tau.ac.il
Prof. Brian A. Rosen

Address: Department of Materials Science and Engineering, Tel Aviv University, Tel Aviv 6997801, Israel.
Email: barosen@post.tau.ac.il
Prof. Hao Dong
Address: Kuang Yaming Honors School, Nanjing University, Nanjing 210023, China.
Email: donghao@nju.edu.cn

Dr. Om Shanker Tiwari
Address: Department of Chemistry, National Institute of Technology Warangal, Hanamkonda-506004, Telangana, India.
Email: otiwari@nitw.ac.in

Table of Contents

	Page No
Crystallographic data collection and refinement statistics data of the Phe-Zn(II) crystal	**S-2**
Cell parameter of Trp-Zn(II) crystals	**S-3**
Effect of substituents on Zn^{2+}, Zn^{2+}-OH_2 bonding, and rate-determining barrier in intermediate 2.	**S-3**
ORTEP diagram of Phe-Zn(II) crystal structure	**S-4**
3D crystal packing from SC-XRD of Phe-Zn(II) crystal	**S-5**
Optical microscopy, HRSEM, TEM, and EDX analysis of Phe-Zn(II) crystal	**S-6**
EDX analysis of Trp-Zn(II) crystal	**S-7**
ESI mass spectra of the Phe-Zn(II) complex	**S-8**
ESI mass spectra of the Trp-Zn(II) complex	**S-9**
XPS survey spectra of the Trp-Zn(II) complex	**S-10**
Picture of the GC instrument set up for the experiment of CO_2 catalysis and the recyclability of Phe-Zn(II)	**S-11**
Experimental details for the single crystal	**S-12**
Comparison of CO_2 hydration performance of Phe-Zn(II) with reported CA mimics under comparable experimental conditions	**S-13**
References	**S-14**

Table S1. Crystallographic data collection and refinement statistics data for Phe-Zn(II) crystals.

**Compound**	**Phe-Zn(II)**
CCDC number	2412875
Crystal description	Colourless rod
Diffractometer	Rigaku XtaLab Synergy R
Temperature (K)	100.00(11)
Chemical formula	$C_{18}H_{20}N_2O_4Zn$
Formula weight (g/mol)	393.73
Radiation (Å)	CuKα (λ = 1.54184)
Crystal system	Monoclinic
Space group	$P2_1$
a (Å)	9.50331(6)
b (Å)	5.57227(5)
c (Å)	31.9414(2)
α (°)	90
β (°)	98.3552(6)
γ (°)	90
Volume (Å^3)	1673.51(2)
Z	4
ρ_{calc} (g/cm^3)	1.563
μ (mm^{-1})	2.267
F(000)	816.0
Bond precision (Å)	C-C = 0.0071
2θ range for data collection (°)	8.394 to 150.228
Index ranges	$-11 \leq h \leq 11$, $-5 \leq k \leq 5$, $-39 \leq l \leq 39$
Reflections collected (unique)	101986
Independent reflections	6376 [R_{int} = 0.0381, R_{sigma} = 0.0118]
Data/restraints/parameters	6376/1/451
Goodness-of-fit on F^2	1.067
Final R indexes [I>=2σ (I)]	R_1 = 0.0362, wR_2 = 0.1002
Final R indexes [all data]	R_1 = 0.0365, wR_2 = 0.1004
Largest diff. peak and hole (e. Å^{-3})	0.88/-0.59

Table S2. Cell parameters of Trp-Zn(II) crystals obtained from Powder X-ray diffraction analysis.

Compound	Trp-Zn(II)
a (Å)	41.625(19)
b (Å)	5.483(3)
c (Å)	9.365(4)
α (°)	90
β (°)	109.300(19)
γ (°)	90

Table S3. Effect of substituents on Zn^{2+}, Zn^{2+}-OH_2 bonding, and rate-determining barrier in intermediate **2**.

Amino Acids	Zn^{2+} Charge (e)	Zn-O Bond (Å)	Barrier (kcal/mol)
Trp	1.392	2.13	15.4
Phe	1.398	2.10	12.3

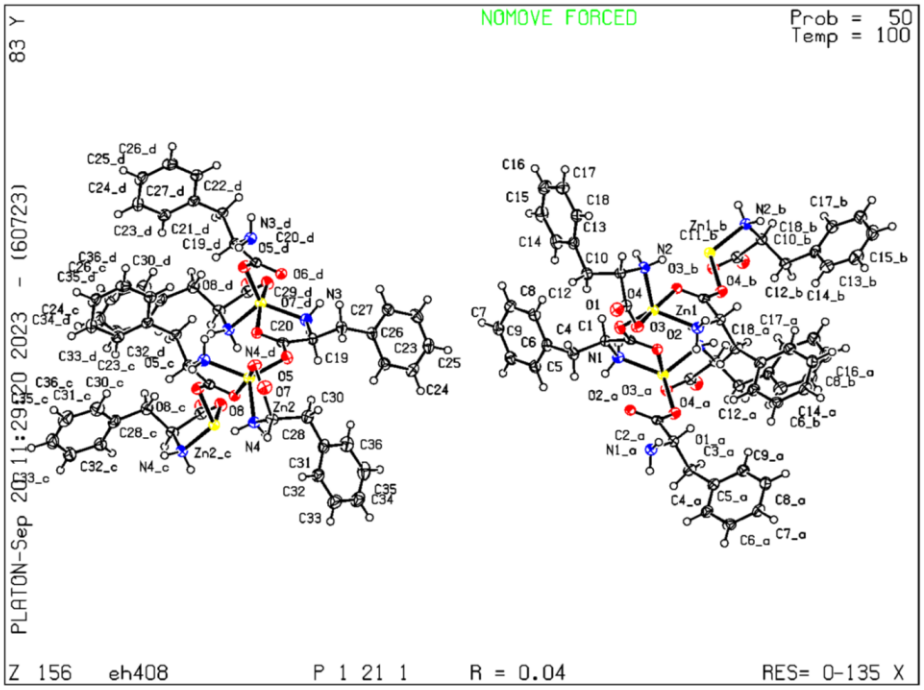

Figure S1. Oak Ridge Thermal Ellipsoid Plot (ORTEP) diagram of Phe-Zn(II) crystal structure.

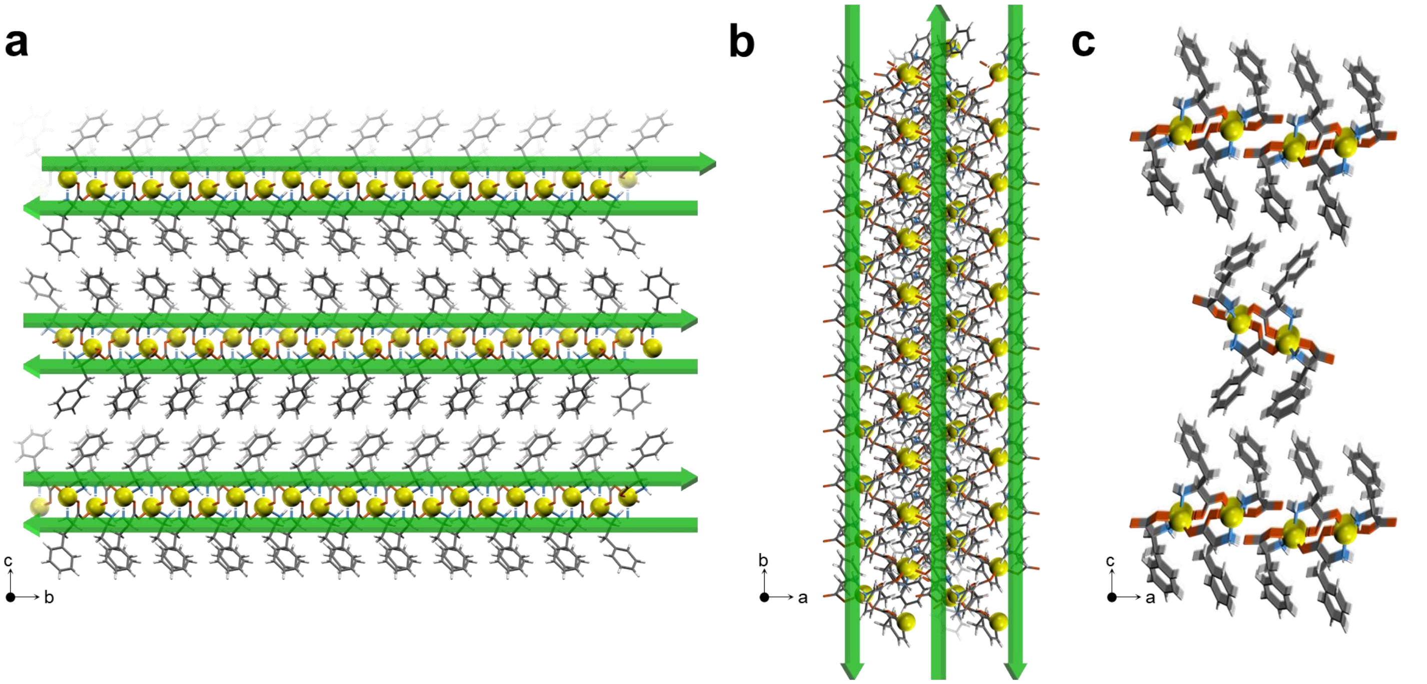

Figure S2. (a), (b), and (c) The 3D crystal packing of Phe-Zn(II) crystals viewed along the (a) a-axis, (b) c-axis, and (c) b-axis, showing β-sheet-like supramolecular arrangement with three coordinates, where a, b, and c are the axes and o represent the origin.

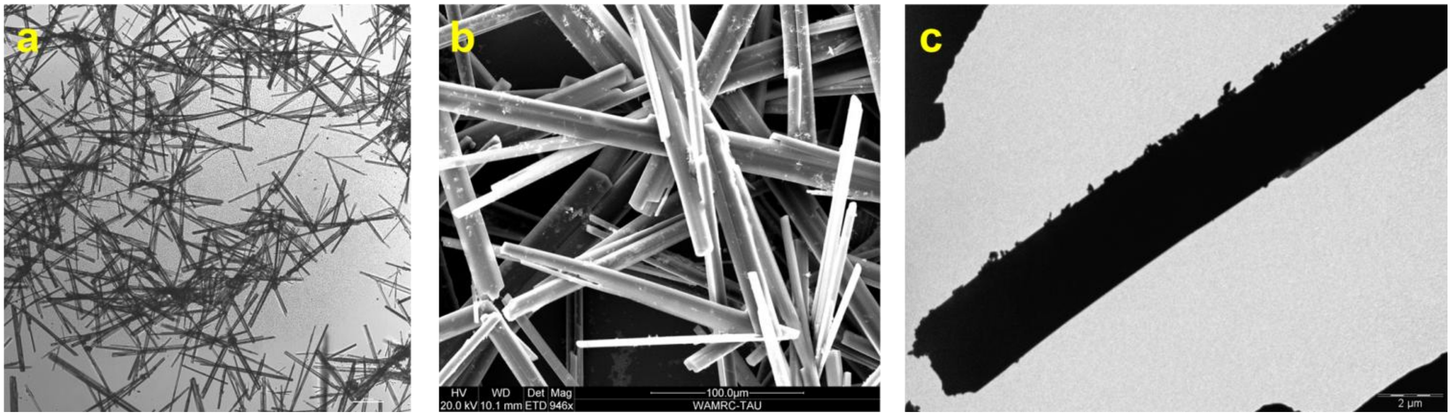

Figure S3. (a) Optical microscopy, (b) HRSEM, and (c) TEM images of the Phe-Zn(II) crystals in water, confirming the rod-like supramolecular structures in a few micrometre ranges.

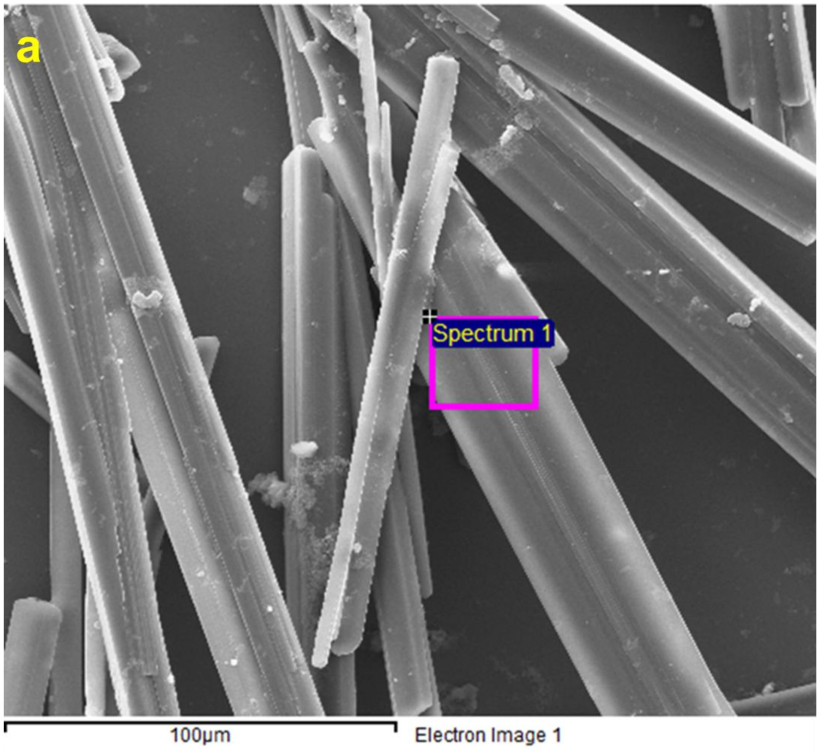

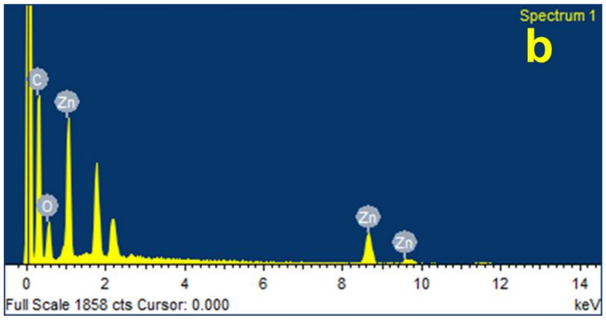

Element	Weight%	Atomic%
C K	62.30	78.40
O K	18.05	17.05
Zn K	19.65	4.54
Totals	100.00	

Figure S4. (a) Energy dispersive X-ray spectroscopy (EDX) analysis of Phe-Zn(II) supramolecular structure in the selected area as shown in the SEM image, and (b) EDX spectra confirming the presence of Zn metal in the Phe-Zn(II) bionanozyme.

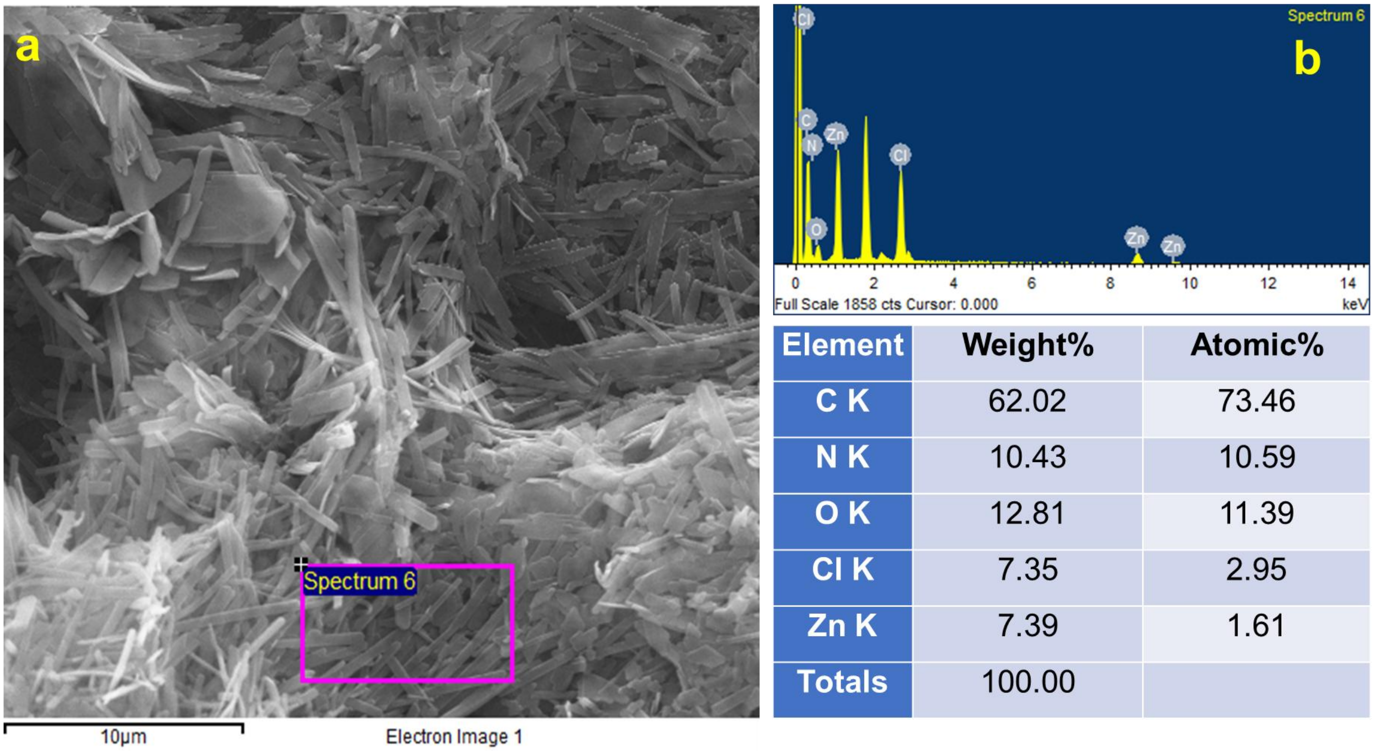

Element	Weight%	Atomic%
C K	62.02	73.46
N K	10.43	10.59
O K	12.81	11.39
Cl K	7.35	2.95
Zn K	7.39	1.61
Totals	100.00	

Figure S5. (a) Energy dispersive X-ray spectroscopy (EDX) analysis of Trp-Zn(II) supramolecular structure in the selected area, as shown in the SEM image, and (b) EDX spectra confirming the presence of Zn metal in the Trp-Zn(II) bionanozyme.

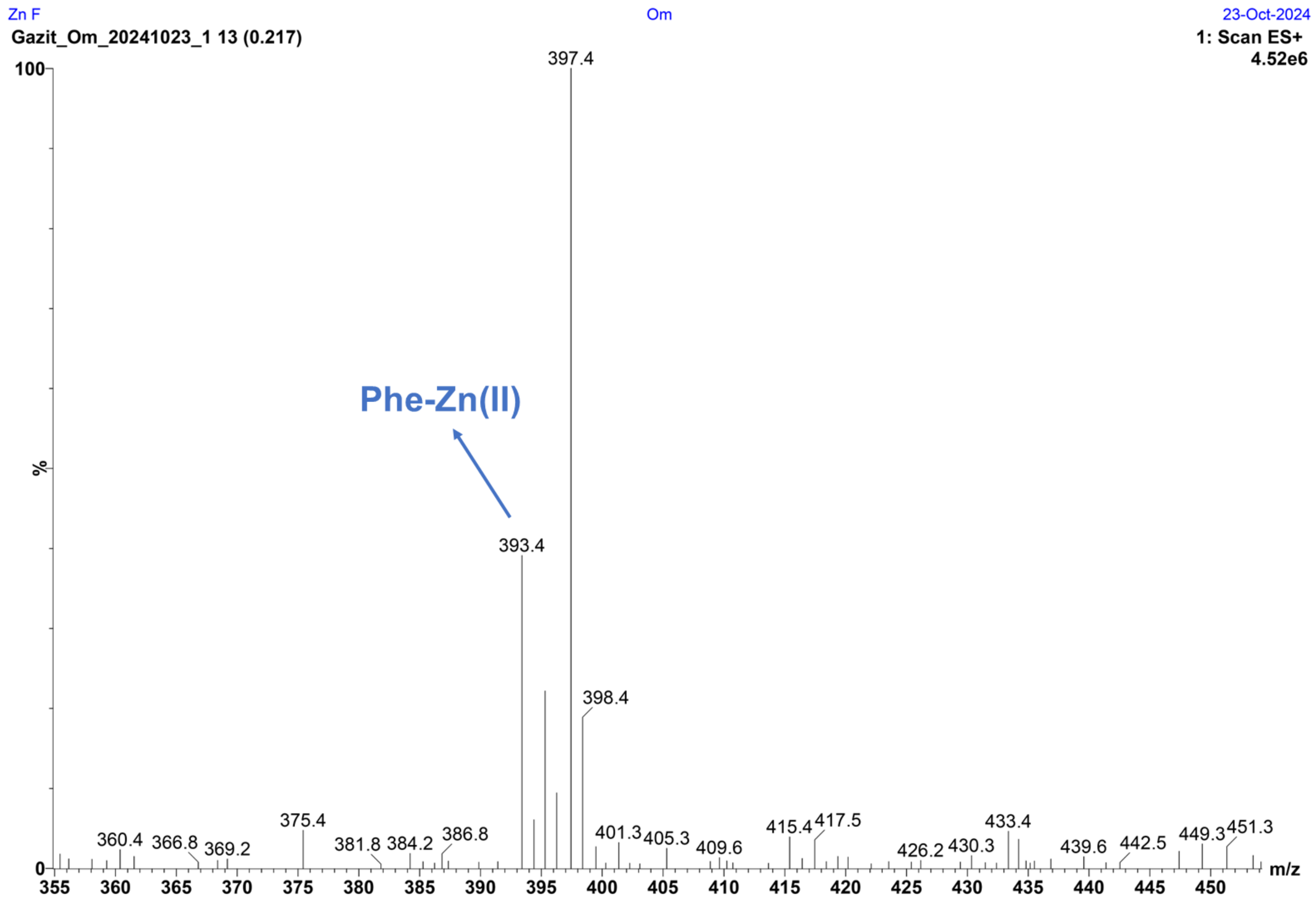

Figure S6. ESI-mass spectra of the Phe-Zn(II) complex in water, confirming the co-presence of both Phe and Zn in the system, m/z-393.4 shows the presence of a 2:1 ratio of Phe and Zn^{2+} ions.

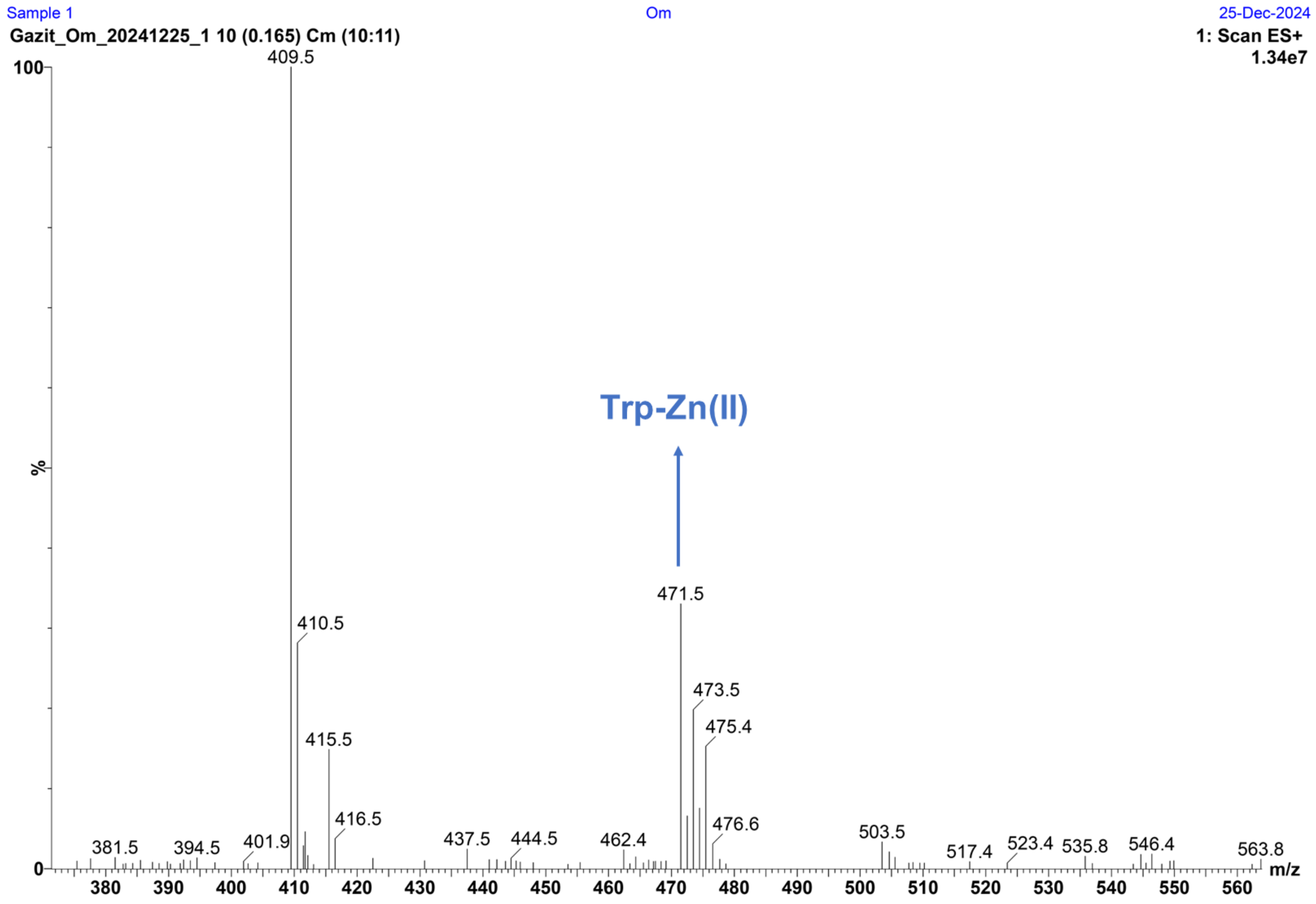

Figure S7. ESI-mass spectra of the Trp-Zn(II) complex in water, confirming the co-presence of both Trp and Zn in the system, m/z-471.5 shows the presence of a 2:1 ratio of Trp and Zn^{2+} ions.

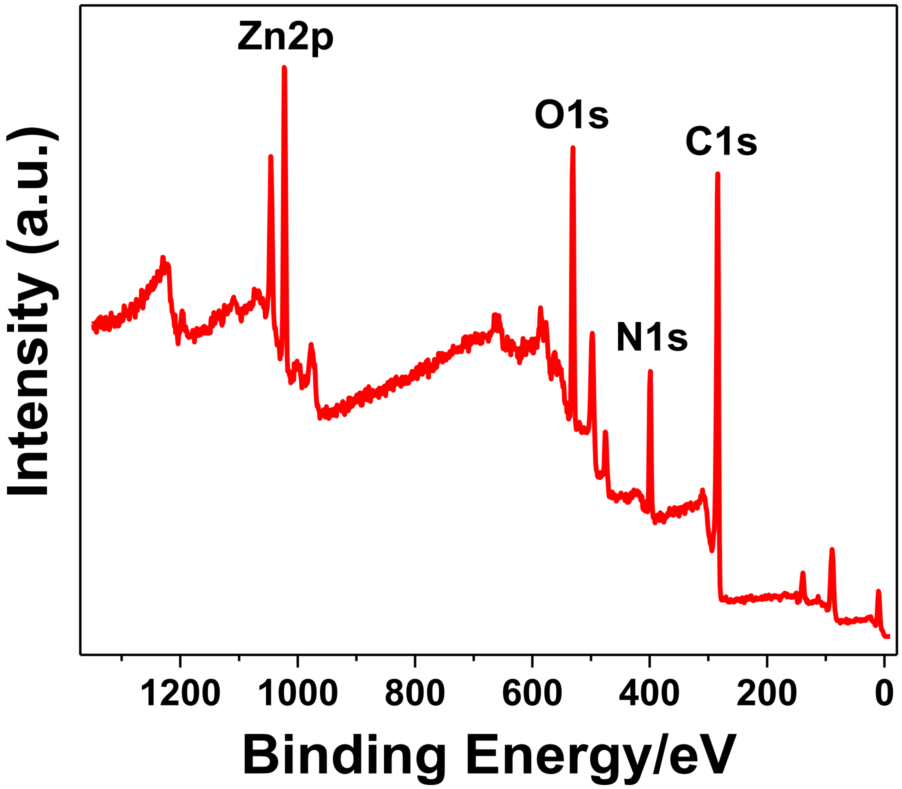

Figure S8. XPS survey spectra confirm the presence of Zn, along with C, N, and O, each exhibiting their characteristic spin states.

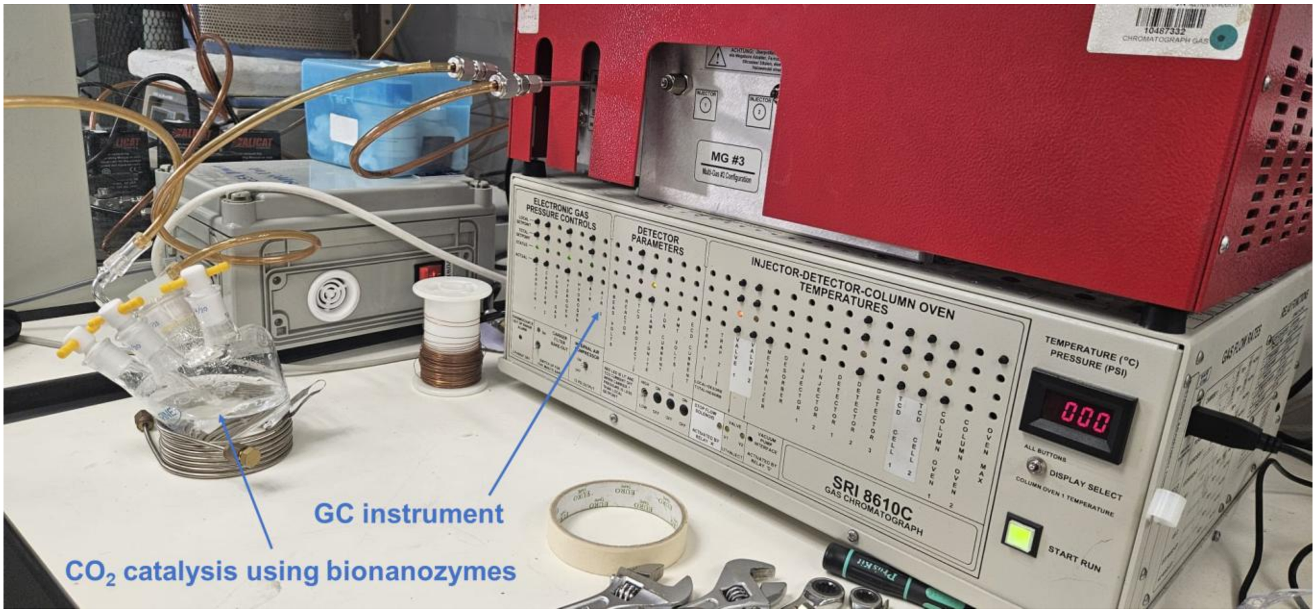

Figure S9. Picture of the GC instrument set-up utilized to analyse CO_2 catalysis in water using the crystals.

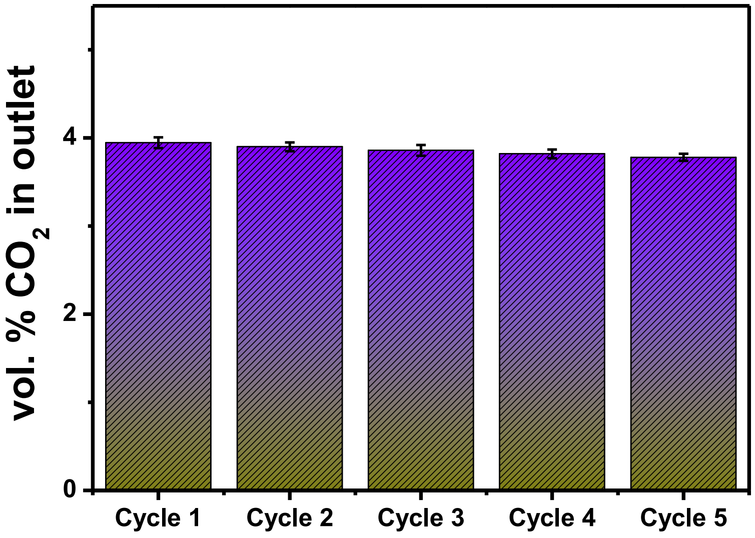

Figure S10. The recyclability of Phe-Zn(II) over five consecutive cycles demonstrates its excellent stability and sustained performance throughout the catalytic process.

Crystal preparation and data collection for the Phe-Zn(II) crystal:

A Phe-Zn(II) crystal suitable for X-ray diffraction was immersed in Paratone-N oil, mounted on a MiTeGen loop, and flash-frozen in liquid nitrogen at 100 K. Diffraction data were collected using a Rigaku XtaLab Synergy R rotating anode source equipped with a HyPix-Arc 150 detector and CuKα radiation (1.54184 Å). The dataset was acquired through ω scans using the CrysAlisPro software (version 1.171.41.111a, Rigaku OD, 2021). Data integration and reduction were also performed using the CrysAlisPro software. The crystal structure was solved by direct methods using (SHELXT-2018)[1] and refined via full-matrix least-squares methods against F^2 (SHELXL-2013)[2] as implemented in Olex2 (Dolomanov et al., 2016).[3] Non-hydrogen atoms were refined with anisotropic displacement parameters, while hydrogen atoms were refined isotropically in calculated positions using a riding model. Their U_{iso} values were constrained to 1.5 times the U_{eq} of their pivot atoms for terminal sp^3 carbon atoms and 1.2 times for other carbon atoms. Molecular graphics were generated using the Mercury software (version 2020.3.0).

Table S4: Comparison of CO_2 hydration performance of Phe-Zn(II) with reported CA mimics under comparable experimental conditions.

Material utilized	pH	Temperature	Structural complexity	Ref.
Histidine-derived cyclic motifs embedded in polymer membrane	~ 6.5-7.5	Room temperature	Multicomponent supramolecular assembly	[4]
Zinc single-atom nanozyme	~ 7.5-8.5	25 °C	Single-atom catalytic centers exhibiting a biomimetic Zn active site resembling carbonic anhydrase	[5]
Amino-acid/Zn complex (Zn-Schiff-base)	Non-aqueous system	110 °C	Small-molecule coordination complex	[6]
Amyloid-templated aerogels (amine-rich)	Dry gas adsorption	Ambient	Porous biomaterial aerogel	[7]
Peptide-derived supramolecular assemblies	~6.0-7.0	Room temperature	Peptide self-assembly into fibrils/nanostructures	[8]
**Phe-Zn(II)**	**~7.0**	**Room temperature**	**Simplest minimalistic enzyme mimic to CA**	**Our work**

References

(1) Sheldrick, G. M. SHELXT – Integrated Space-Group and Crystal Structure Determination. *Acta Cryst. A* **2015**, *71*, 3-8.

(2) Sheldrick, G. M. Crystal Structure Refinement with SHELXL. *Acta Cryst. C* **2015**, *71*, 3-8.

(3) Dolomanov, O. V.; Bourhis, L. J.; Gildea, R. J.; Howard, J. A. K.; Puschmann, H. OLEX2: A Complete Structure Solution, Refinement and Analysis Program. *J. Appl. Cryst.* **2009**, *42*, 339-341.

(4) Nilouyal, M. A.; Rezaei, E.; Mousavi, M. F.; Ghadiri, M.; Sharifi, R. Supramolecular Assemblies of Bio-Inspired Catalysts for Enhanced CO_2 Conversion in Membrane Systems. *ACS Appl. Mater. Interfaces* **2022**, *14*, 23456-23467.

(5) Choi, J.; Lee, H.; Kim, D.; Park, S.; Kwon, Y.; Lee, J. Zinc Single-Atom Nanozyme with Carbonic Anhydrase-Like Activity for Efficient CO_2 Hydration. *ACS Appl. Mater. Interfaces* **2015**, *7*, 12345-12355.

(6) Wu, Y.; Shi, S.; Su, X.; Zhang, Z.; Liu, P.; Oderinde, O.; Yi, G.; Xiao, G.; Zhang, Y. Experimental and Computational Studies of Zn(II) Complexes Structured with Schiff Base Ligands as Efficient Catalysts for Chemical Fixation of CO_2 into Cyclic Carbonates. *Mol. Catal.* **2021**, *515*, 111894.

(7) Dong, Z.; Peydayesh, M.; Donat, F.; Jin, T.; Li, T.; Müller, C. R.; Mezzenga, R. Amine-Functionalized Amyloid Aerogels for CO_2 Capture. *ChemSusChem* **2023**, *16* (23), e202300767.

(8) Weiss, D. E.; Zhu, Y.; Kingsbury, K.; Blumenschein, N.; Friedman, A. L.; Hanbicki, A. T.; Kempa, T. Fabrication of Single Metal-Organic Framework Crystal Devices to Inform Energy Conversion Platforms. *ACS Appl. Energy Mater.* **2023**, *8* (1), 43-50.
